# Discovery of *N*-cyclobutylaminoethoxyisoxazole derivatives as novel sigma-1 receptor ligands with neurite outgrowth efficacy in cells†

**DOI:** 10.1039/c8ra00072g

**Published:** 2018-02-14

**Authors:** Hao Sun, Yun-Jie Wang, Wen-Wen Shi, Fan Yang, Jie Tang, Tao Pang, Li-Fang Yu

**Affiliations:** Shanghai Engineering Research Center of Molecular Therapeutics and New Drug Development, School of Chemistry and Molecular Engineering, East China Normal University 3663 North Zhongshan Road Shanghai 200062 China lfyu@sat.ecnu.edu.cn +86-021-622-31385; Jiangsu Key Laboratory of Drug Screening, State Key Laboratory of Natural Medicines, China Pharmaceutical University Nanjing 210009 PR China tpang@cpu.edu.cn +86-25-832-71043

## Abstract

Herein we reported a series of 14 novel derivatives based on the *N*-cyclobutylaminoethoxyisoxazole scaffold. *In vitro* binding studies of these compounds demonstrated their low nanomolar to subnanomolar potencies as σ1 receptor ligands, with moderate to excellent selectivity over the σ2 receptor as represented by compounds 17–30. The majority of the derivatives scored high (>4.7) in the CNS MPO appraisal system, indicating their high likelihood in penetrating the blood–brain barrier. A number of these compounds exhibited significant neurite outgrowth efficacy in N1E-115 neuronal cells and displayed excellent selectivity for σ1 receptors over the selected endogenous neurotransmitter transporters, such as DAT, NET and SERT. Among the mini-series, compound 28 (*K*_i_ σ1 = 0.2 nM, *K*_i_ σ2 = 198 nM, CNS MPO score = 5.4) emerged as a promising selective σ1 receptor ligand that warrants its further evaluation as a potential therapeutic for neurodegenerative diseases.

## Abstract 2

Herein we reported a series of 14 novel derivatives based on the *N*-cyclobutylaminoethoxyisoxazole scaffold.

## Graphical abstract

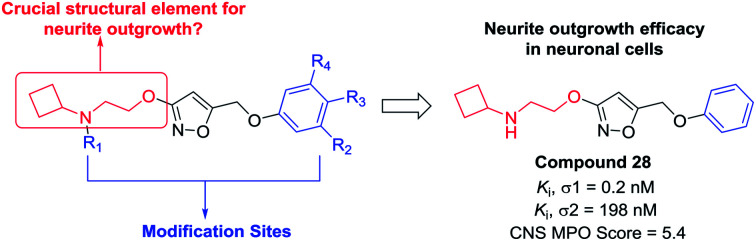

## Introduction

Neurodegenerative diseases, such as Alzheimer's disease (AD), Parkinson's disease (PD), amyotrophic lateral sclerosis (ALS) and Huntington's disease (HD) are caused by the progressive loss of neuronal integrity or acute neuron injury such as stroke or trauma in the brain and spinal cord.^1–3^ Current therapeutic strategies for neurodegenerative diseases are mainly aimed to decrease CNS neuron damage or brain dysfunction by conferring neuroprotection and neurogenesis.^4,5^ Consequently, the development of effective therapeutic medicine or discovery of new biological targets with neurogenesis activities remains an urgent need in the treatment of neurodegenerative diseases.

During the past decades, various kinds of receptors, ion channels and signaling pathways associated with neurogenesis activities have been identified as potential therapeutic targets for neurodegenerative diseases. Among these, the σ1 receptor has attracted wide attention.^6^ σ1 receptor is one of the subtype belonging to the σ receptors family which was first discovered by Martin in 1976.^7^ The σ1 receptor has been cloned and encoded as a protein of 223 amino acids, and specifically localized at the mitochondrial-associated endoplasmic reticulum membrane.^8,9^ In 2016, the crystal structure of the human σ1 receptor was reported in complex with two divergent ligands. The structure indicated that the human σ1 receptor has a trimeric architecture with a single transmembrane domain.^10^ The literature reports to date indicate that σ1 receptor is considered as chaperonin to modulate signaling pathways of ER–mitochondrion such as Ca^2+^, K^+^, and NMDA and IP_3_ receptors, playing an important role in healthy CNS functioning.^11–13^ In particular, neurogenesis efficacy of brain penetrant σ1 receptor ligands is of a considerable interest, which has been attributed to the σ1 receptor's role in the modulation of cellular trafficking.^14,15^ The activation of σ1 receptor leads to the transfer of cholesterol, ceramides and essential amino acids that are essential for the growth and proliferation of the neurons, along with an increased gene expression of specific protein and synthesis of various growth factors involved in neuronal outgrowth.^16–18^ Furthermore, neurogenesis function was reported to be subdued in the hippocampus of σ1 receptor-knockout animals.^19^

**Fig. 1:**
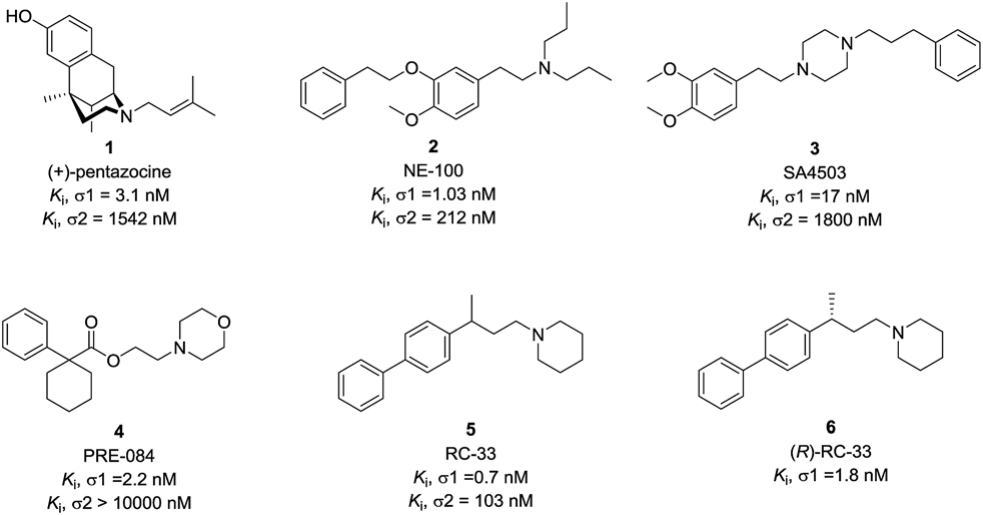
Representative σ1 receptor ligands.

An increasing number of σ1 receptor ligands that stimulate neurite outgrowth in cellular screening models have been reported to date. Pentazocine (1) is a classical σ1 receptor ligand, capable of enhancing the neurite outgrowth in PC-12 cellular model caused by the activation of σ1 receptor and this activity was antagonized by NE-100 (2), a σ1 receptor antagonist.^20^ Ruscher *et al.* reported that the cortical neurons outgrowth upon treatment with SA4503 (3) could be prevented with σ1 receptor silencing.^16^ In addition, some reports in the literature suggested that the neuronal outgrowth activity by σ1 receptor activation is synergistic with some growth factors. PRE-084 (4) is an example of high affinity σ1 receptor ligand that can enhance neurite outgrowth and elongation induced by NGF or EGF in PC12 cells.^21^ Subsequently, PRE-084 could enhance the survival of the motoneurons and improve locomotor function in murine models of ALS.^22^ RC-33 (5), as a σ1 receptor ligand, also exhibited the neurite outgrowth effects and good *in vitro* metabolic stability.^23–25^ The (*R*)-enantiomer of RC-33 (6) has been selected as a lead compound for studies in multiple sclerosis.^26^ Therefore, the development of novel, brain-penetrant σ1 receptor ligands with neurogenesis efficacy could find some therapeutic use for neurodegenerative diseases (Fig. 1).

**Fig. 2:**
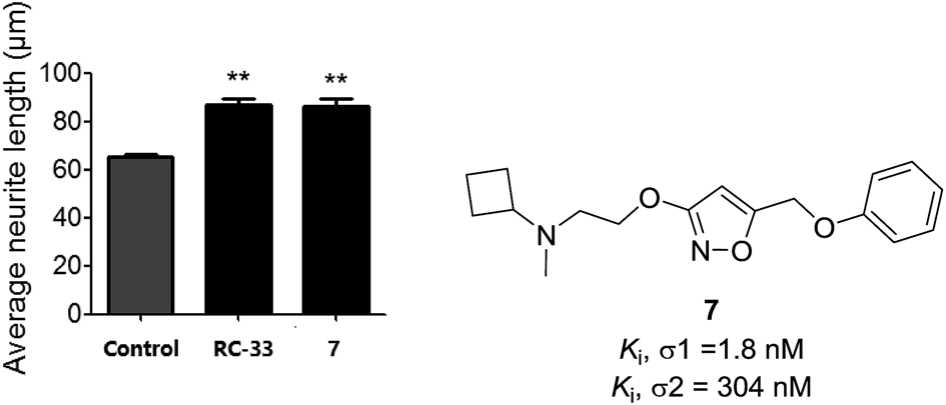
The potential effect of compound 7 (10 μM) and RC-33 (10 μM) on NGF (2.5 ng mL^−1^)-induced neurite outgrowth model in PC12 cells. ^a^See Experimental section. Each histograms represent the mean ± SEM of at least five different experiments. Statistically significant differences: **p < 0.01 *vs.* control using NGF alone.

In our preliminary work in the σ receptors drug discovery, we developed a series of alkoxyisoxazole derivatives that were found to be σ1 receptor ligands with high affinity and selectivity over the σ2 receptor.^27^ Based on this work,^27^ we further screened some of the most potent derivatives in a cellular model of neurite outgrowth. During the course of this investigation, alkoxyisoxazole 7 exhibited synergistic neurite outgrowth effects at a level similar to that of the positive control RC-33 (Fig. 2). Encouraged by this finding, we performed further structure–activity relationship (SAR) studies on this scaffold, incorporating *N*-cyclobutylaminoethoxy linker that was hypothesized to be a crucial structural element for neurite outgrowth. With 7 as the starting compound, herein we reported our continued efforts in the SAR studies of the *N*-cyclobutylaminoethoxyisoxazole as σ1 receptor ligands and their cellular evaluation in neurite outgrowth assay.

## Results and discussion

### Chemistry

**Scheme 1:**
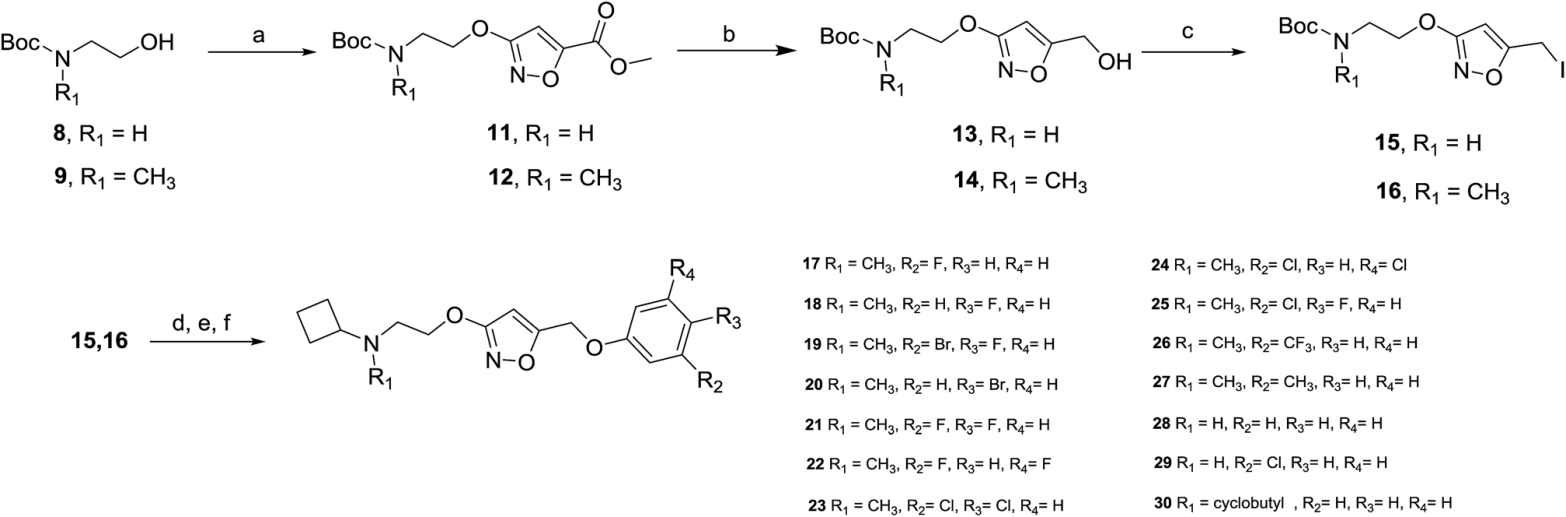
^a^Reagents and conditions: (a) methyl 3-hydroxyisoxazole-5-carboxylate (10), diisopropyl azodicarboxylate, PPh_3_, THF, 0 °C to rt; (b) LiBH_4_, THF, 0 °C to rt; (c) I_2_, PPh_3_, imidazole, CH_2_Cl_2_, 0 °C to rt; (d) phenol, or substituted phenol, K_2_CO_3_, DMF, rt; (e) HCl/EtOAc, rt; (f) cyclobutanone, Na(CN)BH_3_, CH_3_CO_2_H, CH_3_OH, rt.

The synthesis of the new compounds was accomplished as shown in Scheme 1. The starting material 8 or 9 underwent a Mitsunobu reaction with previously reported methyl 3-hydroxyisoxazole-5-carboxylate (10) to obtain the intermediate esters 11 and 12. Lithium borohydride reduction afforded the alcohols 13 and 14, respectively, which were converted to the corresponding iodides 15 and 16 using iodine, triphenylphosphine and imidazole as a base. Subsequent reaction of the iodides with phenol or substituted phenols under alkaline conditions gave the Boc-protected isoxazole intermediates. Finally, treatment with hydrogen chloride and reductive amination with cyclobutanone and sodium cyanoborohydride resulted in final compounds 17–30. All final compounds were recrystallized by HCl/ethyl acetate as hydrochloride salts.

### Radioligand binding studies at σ1 and σ2 receptors.^28^

In this study, [^3^H] pentazocine and [^3^H]DTG (6) were used as the radioligands at σ1 and σ2 receptors. The binding affinity assays of compounds 17–30 are shown in Table 1. Firstly, we examined the effects of changing the substituents on the benzene ring. 3-Methyl substituted compound 27 retained high affinity to parent compound 7 at σ1 receptor but a 3-fold increase in the binding affinity for σ2 receptor. Compound 26 with a 3-trifluoromethyl substituent showed similar affinity for σ1 receptor and significantly decreased selectivity over the σ2 receptor when compared with compound 7. Mono-halogen substitution on the benzene ring exemplified by compounds 17–20 generally retained affinity for σ1 receptor compared with compound 7. However, all of these mono-halogen substituted derivatives also exhibited subdued selectivity over the σ2 receptor. Notably, the 3-bromo substituted compound 19 displayed a better selectivity (*K*_i_ σ2/*K*_i_ σ1 = 131) than the 4-bromo substituted compound 20. The derivatives 21–25 also maintained similar high affinities for the σ1 receptor upon incorporation of two halogen atoms on the benzene ring. However, this is also accompanied with higher binding affinity to the σ2 receptor. Among the disubstituted compounds, 3,5-dichloro substituted compound 24 showed a 4-fold decreased affinity for σ1 receptor and more than 10-fold increased binding affinity for σ2 receptor. Next, we examined the effects of removing the methyl group on the basic nitrogen atom. Gratifyingly, compound 28 showed a high affinity for σ1 receptor (*K*_i_ σ1 = 0.2 nM) and excellent selectivity on σ2 receptor (*K*_i_ σ2 = 198 nM). Compound 29 with a 3-chloro substituted benzene ring also exhibited high binding affinity for σ1 receptor (*K*_i_ σ1 = 0.4 nM) but slightly lower selectivity over σ2 receptor (*K*_i_ σ2 = 64.3 nM). Compound 30 was a by-product obtained during the synthesis of compound 28, which showed a decreased binding affinity and selectivity indicating an unfavourable steric repulsion by the cyclobutyl group. In summary, except for compound 30, most of the derivatives exhibited high binding affinities for σ1 receptor (*K*_i_ σ1 < 10 nM). However, all the benzene ring substituted derivatives showed various degree of decreased selectivity over the σ2 receptor, implying that the non-substituted benzene ring was a crucial structural element for selectivity. The results for the secondary amines 28 and 29 indicate that the hydrogen bond donor on basic nitrogen atom was beneficial for increased binding affinity to the σ1 receptor.

**Table 1:** Binding affinities of derivative at σ1 and σ2 receptors^a^

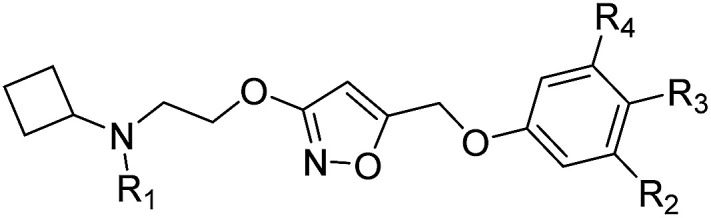							
Compound	R_1_	R_2_	R_3_	R_4_	*K*_i_ σ1 (nM)	*K*_i_ σ2 (nM)	Selectivity (*K*_i_ σ2/*K*_i_ σ1)
7^b^	–CH_3_	–H	–H	–H	1.8 ± 0.2	307 ± 56	171
17	–CH_3_	–F	–H	–H	1.9 ± 0.0	91.5 ± 7.5	48
18	–CH_3_	–H	–F	–H	0.9 ± 0.1	78.5 ± 19.5	87
19	–CH_3_	–Br	–H	–H	0.8 ± 0.1	105 ± 21	131
20	–CH_3_	–H	–Br	–H	5.5 ± 1.3	86.6 ± 16.8	16
21	–CH_3_	–F	–F	–H	1.7 ± 0.3	153 ± 37	90
22	–CH_3_	–F	–H	–F	2.4 ± 1.2	57.0 ± 20.1	24
23	–CH_3_	–Cl	–Cl	–H	3.2 ± 1.6	36.0 ± 9.7	11
24	–CH_3_	–Cl	–H	–Cl	7.8 ± 3.2	22.8 ± 11.1	3.0
25	–CH_3_	–Cl	–F	–H	1.3 ± 0.4	32.0 ± 6.0	25
26	–CH_3_	–CF_3_	–H	–H	4.0 ± 1.2	38.0 ± 2.0	9.5
27	–CH_3_	–CH_3_	–H	–H	4.2 ± 1.9	124 ± 68	29.5
28	–H	–H	–H	–H	0.2 ± 0.0	198 ± 64	990
29	–H	–Cl	–H	–H	0.4 ± 0.1	64.5 ± 13.2	161
30	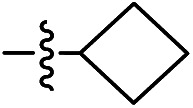	–H	–H	–H	16.0 ± 5.0	53.5 ± 0.5	3.3

a See Experimental section. Radioligands: σ1: [^3^H]-(+)-pentazocine; σ2: [^3^H] DTG (ditolylguanidine). Evaluation platform: σ1: Guinea pig homogenate; σ2: PC12 cells.

b The *K*_i_ values for compound 7 are cited from the literature.^27^ Data are expressed as the mean ± SEM and each performed from three independent experiments.

### Blood–brain barrier permeability studies by CNS MPO

In this study, all of the novel σ1 receptor ligands were tested their blood–brain barrier permeability by using the central nervous system multiparameter optimization (CNS MPO) desirability tool which was reported by Travis T. Wager *et al.*^29,30^ In CNS MPO appraisal system, six structural properties of candidates would be used such as Clog *P*, Clog *D*, MW, TPSA, HBDs and p*K*_a_ that were determined as important factors in the BBB permeability. Each property was valued between 0 to 1 and weighted equally. Final collective score ranged from 0 to 6, with the higher scores of CNS MPO correlating with desirable brain permeability. Through the analysis of CNS MPO scores on more than hundreds of the drugs and original candidates acting on the CNS, a majority of them had CNS MPO desirability scores greater than 4.^30^ As showed in Table 2, majority of the newly synthesized alkoxyisoxazoles obtained CNS MPO scores greater than 4. In particular, compound 28 scored the highest CNS MPO scores (5.4), in addition to the observed high binding affinities at σ1 receptor (Table 1).

**Table 2:** CNS MPO scores of compound 7 and all the derivatives^a^

Compound	Clog *P*	Clog *D*	MW	TPSA	HBDs	p*K*_a_	CNS MPO scores^b^
7	3.27	2.08	302.37	43.29	0	8.89	5.4
17	3.55	2.18	320.36	43.29	0	8.89	5.2
18	3.44	2.17	320.36	54.15	0	8.94	5.3
19	4.27	2.17	381.07	43.29	0	8.89	4.7
20	4.27	3.11	381.27	43.29	0	8.89	4.2
21	3.68	2.22	338.35	43.29	0	8.89	5.1
22	3.75	2.33	338.35	43.29	0	8.89	5.0
23	4.77	3.47	371.26	43.29	0	8.89	3.9
24	4.89	3.75	371.26	43.29	0	8.89	3.7
25	4.32	3.15	354.80	43.29	0	8.89	4.3
26	4.40	3.14	370.37	43.29	0	8.89	4.2
27	3.77	2.53	316.18	43.29	0	8.89	4.9
28	2.68	0.86	288.15	52.08	1	8.94	5.4
29	3.52	1.68	322.11	52.08	1	8.94	5.2
30	3.64	1.97	342.19	43.29	0	8.75	5.3

a The data sources and explanations. Clog *P*: calculated partition coefficient; Clog *D*: calculated distribution coefficient at pH 7.4; MW: molecular weight; TPSA: topological polar surface area; HBDs: number of hydrogen-bond donors: p*K*_a_: most basic center. The calculated physicochemical properties of the derivatives were obtained using standard commercial software: ACD/Laboratories, version 6.0 for Clog *P*, p*K*_a_, HBDs and Clog *D* at PH 7.4, ChemBioDraw, version 14.0 for MW and TPSA.

b Last CNS MPO scores were calculated by the tools reported by Travis T. Wager *et al.*^30^

### Neuritogenesis studies in N1E-115 cells

**Fig. 3:**
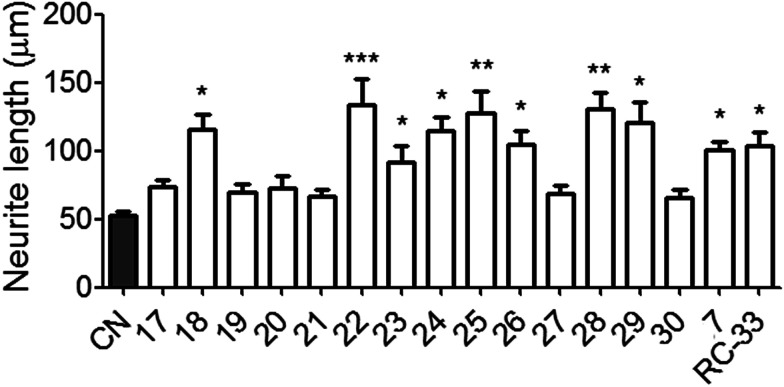
Neurite outgrowth effects of all derivatives in N1E-115 neuronal cells. ^a^See Experimental section. Each histograms represent the mean ± SEM of at least five different experiments. Statistically significant differences: *p < 0.05, **p < 0.01, ***p < 0.001 *vs.* control (CN) using DMSO alone.

Following the radioligand binding studies and CNS MPO calculations, all of the synthesized derivatives were further tested in cellular neuritogenesis studies. In this study, N1E-115 neuronal cells were used as a screening platform for assessing the neuritogenesis effects of candidate compounds.^31,32^ All the derivatives and positive control (compound 7 and RC-33) were assessed at 10 μM concentration in DMSO, and vehicle group using DMSO alone. The test results are shown in Fig. 3. Gratifyingly, a large proportion of the drug administration significantly increased the average neurite length of N1E-115 cells at 10 μM concentration *vs.* vehicle group, suggestive of a neuritogenesis effect. Through the analysis of test results, most of the disubstituted derivatives such as compound 22–25 exhibited greater neuritogenesis efficacy when compared with vehicle control. The 4-fluoro substituted compound 18 and 3-trifluoromethyl substituted compound 26 displayed better efficacy than other mono-substituted compounds. The higher efficacies observed for compounds 28 and 29 indicated that the secondary amine moiety was beneficial for promoting neuritogenesis efficacy within this series. Particularly, we obtained six compounds (18, 22, 24, 25, 28 and 29) that showed preferable neurite outgrowth effects compared to the parent compound 7 and positive control RC-33 in N1E-115 neuronal cells.

### Selectivity studies at selected neurotransmitter transporters

In our previous work, we found that some derivatives based on the alkoxyisoxazole scaffold not only bind to σ1 receptor but also to common neurotransmitter transporters, such as DAT, NET and SERT.^27,33^ In order to exclude the potential interference of other receptor pathways to the observed neuritogenesis efficacy, we assessed the binding affinities of compounds 7, 18, 22, 24, 25, 28 and 29 at the biogenic amine transporters (NET, DAT, and SERT). Gratifyingly, no appreciable binding affinity was observed for these transporters using 10 μM concentration of tested compounds. This set of results indicated that the selected compounds had excellent selectivity for σ1 receptor over DAT, NAT and SERT (Table 3).

**Table 3:** Binding affinities of selected compounds at NET, DAT, and SERT^a^

Compound	*K*_i_, DAT (nM)	*K*_i_, NET (nM)	*K*_i_, SERT (nM)	*K*_i_, σ1 (nM)
GBR12909	8.8 ± 3.8^b^	—	—	—
Desipramine	—	2.5 ± 0.4	—	—
Amitriptyline	—	—	5.4 ± 0.9	—
7	NA^c^	NA	NA	1.8 ± 0.2
18	5631^d^	NA	NA	0.9 ± 0.1
22	>10 000	2878	NA	2.4 ± 1.2
24	1811	1606	NA	7.8 ± 3.2
25	>10 000	1780	NA	1.3 ± 0.4
28	NA	7102.5	NA	0.2 ± 0.0
29	1518	600.5	NA	0.4 ± 0.1

a See Experimental section. DAT: dopamine transporter; NET: norepinephrine transporter; SERT: serotonin transporter. Radioligands: DAT: [^3^H] WIN35428; NET: [^3^H] nisoxetine; SERT: [^3^H] citalopram.

b The *K*_i_ values for GBR12909, desipramine, amitriptyline and compound 7 are cited from the literature.^27^

c NA: not active, defined as <50% binding in the primary assay at 10 μM. *K*_i_ values were determined for those targets where the binding efficacy at 10 μM was greater than 50%.

d Data are expressed as average of *K*_i_ values and each performed from three independent experiments.

## Conclusions

In summary, 14 novel derivatives based on the *N*-cyclobutylaminoethoxyisoxazole scaffold were designed and synthesized. Most of the derivatives showed potent binding affinities to the σ1 receptor (*K*_i_ < 10 nM) and good selectivity over the σ2 receptor in radioligand binding tests. CNS MPO scores were calculated for predicting their blood–brain barrier permeability and the results showed most of derivatives obtained favourable CNS MPO scores greater than 4. All of the compounds were assessed for their neurite outgrowth efficacy in N1E-115 cells. Compounds 18, 22, 24, 25, 28 and 29 exhibited significant neuritogenesis effects at 10 μM concentration *vs.* vehicle group and superior efficacy of increasing the average neurite length compared with compound 7 and RC-33. Moreover, compounds 18, 22, 24, 25, 28 and 29 showed great selectivity for σ1 receptor over DAT, NET, and SERT transporters. Among these derivatives, compound 28 was found as the most promising compound with excellent *in vitro* binding profile (*K*_i_ σ1 = 0.2 nM, *K*_i_ σ2 = 198 nM), high CNS MPO score (5.4) and significant neuritogenesis efficacy, which will be further developed as potential therapeutics for neurodegenerative diseases.

## Experimental section

### Chemistry

#### General methods

Unless otherwise specified, commercial reagents and solvents were all of analytical grade or of chemical purity (>99%). Anhydrous THF was obtained by distillation over sodium wire, respectively. All reactions were run under a nitrogen atmosphere and all reaction vessels were oven-dried. The TLC was performed on silica gel GF254. Column chromatographic purification was carried out using silica gel (200–300 mesh). ^1^H and ^13^C NMR spectra were recorded on a Bruker Advance III 400 spectrometer at 400 MHz (^1^H) and 101 MHz (^13^C). Chemical shifts are reported in *δ* (ppm) using the *δ* 0 signal of tetramethylsilane (TMS) as internal standards. High resolution mass spectra were performed using a Bruker ESI-TOF high-resolution mass spectrometer. Purities of final compounds were established by Agilent 1200 HPLC system with a ZORBAX Eclipse XDB-C18 column, with detection at 220 or 254 nm on a variable wavelength detector G1365D; flow rate = 1.4 mL min^−1^; gradient of 0 to 100% methanol in water (both containing 0.05 vol% of TFA) in 25 min.

#### General procedure for the Mitsunobu reaction (method A)

Diisopropyl azodicarboxylate (2.0 mmol) was added dropwise into a stirred solution of 8 or 9 (1.0 mmol), 10 (1.2 mmol), and PPh_3_ (2.0 mmol) in anhydrous THF (20 mL) at 0 °C under the nitrogen. After stirring overnight at rt, the solvent was evaporated, and the residue was dissolved in EtOAc (30 mL). The organic layer was washed with water (20 mL) and brine (15 mL), dried over Na_2_SO_4_ and evaporated *in vacuo*. The residue was purified by flash chromatography to give the product.

#### General procedure for the reduction reaction (method B)

LiBH_4_ (4 mmol) was added into a solution of compound 11 or 12 (0.8 mmol) in anhydrous THF (20 mL) with ice cooling under nitrogen. After stirring overnight at rt, the reaction was quenched by saturated aqueous NH_4_Cl solution with ice cooling. The mixture was extracted with EtOAc (3 × 30 mL). The organic layer was washed with water (20 mL) and brine (15 mL), dried over Na_2_SO_4_ and evaporated *in vacuo*. The residue was purified by flash chromatography to give the product.

#### General procedure for the preparation of iodides (method C)

I_2_ (1.2 mmol) was added into a stirred solution of compound 13 or 14 (0.7 mmol), imidazole (2.0 mmol), and PPh_3_ (1.2 mmol) in anhydrous DCM (10 mL) with ice cooling under nitrogen. After reacting completely at rt, the solvent was evaporated. The residue was purified by the flash chromatography to give the product.

#### General procedure for the preparation of phenyl ethers (method D)

K_2_CO_3_ (6.0 mmol) was added into a stirred solution of compound 15 or 16(1.0 mmol) and phenol or substituted phenol (2.0 mmol) in anhydrous DMF (4 mL) under nitrogen. After stirring overnight at rt, water (30 mL) was added. The mixture was extracted with EtOAc (2 × 30 mL), and the combined organic layers were washed with water (3 × 20 mL) and brine (20 mL), dried over Na_2_SO_4_ and evaporated *in vacuo*. The residue was purified by the flash chromatography to give the phenyl ether product.

#### General procedure for the deprotection (method E)

HCl/EtOAc (4 mol L^−1^, 2 mL) was added into a stirred solution of the *N*-Boc protected intermediates (1.0 mmol) in CH_2_Cl_2_ (5 mL) under nitrogen with ice cooling. The mixture was stirred overnight at rt. After the solvent was evaporated, the residue was by ether (20 mL). The resulting solid was filtered to give the HCl salt.

#### General procedure for the reductive amination (method F)

NaCNBH_4_ (0.6 mmol) was added into a stirred solution of compound primary or secondary amine (0.4 mmol), cyclobutanone (0.5 mmol) and acetic acid (0.05 mL) in CH_3_OH (10 mL) at rt under nitrogen. After stirring overnight at rt, the solvent was evaporated and the residue was extracted with EtOAc (3 × 10 mL). The combined organic layer was washed with water (20 mL) and brine (15 mL), dried over Na_2_SO_4_ and evaporated *in vacuo*. The residue was purified by flash chromatography. To a solution of the *N*-alkyl compound in CH_2_Cl_2_ (3 mL) was added HCl/EA (4 mol L^−1^, 1 mL) under N_2_ with ice cooling. The mixture was stirred overnight at rt. The solvent was evaporated to give the HCl salt.

#### Methyl 3-hydroxyisoxazole-5-carboxylate (10)

Dimethyl 2-butynedioate was added into a solution of *N*-hydroxyurea (0.05 mol) and DBU (0.06 mol) in methanol (50 mL) at 0 °C under nitrogen. The reaction was stirred at 0 °C for 1 h. After stirred overnight at rt, the solvent was evaporated *in vacuo* and the residue was dissolved into water (20 mL) and acidified to pH 1 with 1 N hydrochloric acid. The mixture was extracted with diethyl ether (3 × 25 mL), dried over Na_2_SO_4_ and evaporated *in vacuo*. The resulting solid was recrystallized from chloroform to give the product. White solid; yield 41%. ^1^H NMR (400 MHz, DMSO-*d*_6_) *δ* 11.89 (s, 1H), 6.75 (s, 1H), 3.87 (s, 3H).

#### Methyl 3-(2-((*tert*-butoxycarbonyl)amino)ethoxy)isoxazole-5-carboxylate (11)

This compound was obtained from compounds 8 and 10 employing method A. White solid, yield 85%. ^1^H NMR (400 MHz, CDCl_3_) *δ* 6.55 (s, 1H), 4.35 (t, *J* = 5.0 Hz, 2H), 3.95 (s, 3H), 3.63–3.46 (m, 2H), 1.45 (s, 9H). ^13^C NMR (101 MHz, CDCl_3_) *δ* 171.3, 160.4, 157.0, 155.7, 100.7, 77.2, 69.7, 52.9, 39.7, 28.3.

#### Methyl 3-(2-((*tert*-butoxycarbonyl)(methyl)amino)ethoxy)isoxazole-5-carboxylate (12)

This compound was obtained from compounds 9 and 10 employing method A. White solid; yield 91%. ^1^H NMR (400 MHz, CDCl_3_) *δ* 6.54 (s, 1H), 4.41 (m, 2H), 3.95 (s, 3H), 3.63 (m, 2H), 2.94 (s, 3H), 1.45 (s, 9H). ^13^C NMR (101 MHz, CDCl_3_) *δ* 171.3, 160.3, 157.0, 155.5, 100.7, 79.8, 68.6, 52.8, 47.8, 35.3, 28.3.

#### *tert*-Butyl(2-((5-(hydroxymethyl)isoxazol-3-yl)oxy)ethyl)carbamate (13)

This compound was obtained from 11 employing method B. Colorless oil; yield 90%. ^1^H NMR (400 MHz, CDCl_3_) *δ* 5.89 (s, 1H), 4.94 (s, 1H), 4.64 (s, 2H), 4.28 (t, *J* = 5.0 Hz, 2H), 3.56–3.47 (m, 2H), 2.48 (br, 1H), 1.44 (s, 9H). ^13^C NMR (101 MHz, CDCl_3_) *δ* 173.0, 171.5, 156.0, 93.1, 79.8, 69.0, 56.5, 39.8, 28.3.

#### *tert*-Butyl(2-((5-(hydroxymethyl)isoxazol-3-yl)oxy)ethyl)(methyl)carbamate (14)

This compound was obtained from 12 employing method B. Colorless oil; yield 88%. ^1^H NMR (400 MHz, CDCl_3_) *δ* 5.81 (s, 1H), 4.55 (s, 2H), 4.25 (t, *J* = 5.2 Hz, 2H), 3.57 (br, 1H), 3.52 (t, *J* = 5.2 Hz, 3H), 2.85 (s, 3H), 1.37 (s, 9H). ^13^C NMR (101 MHz, CDCl_3_) *δ* 172.0, 170.5, 154.7, 92.2, 79.0, 67.0, 55.7, 47.0, 34.2, 27.1.

#### *tert*-Butyl(2-((5-(iodomethyl)isoxazol-3-yl)oxy)ethyl)carbamate (15)

This compound was obtained from 13 employing method C. Yellow oil; yield 57%. ^1^H NMR (400 MHz, CDCl_3_) *δ* 5.92 (s, 1H), 4.91 (s, 1H), 4.28 (m, 2H), 4.26 (s, 2H), 3.54–3.47 (m, 2H), 1.44 (s, 9H). ^13^C NMR (101 MHz, CDCl_3_) *δ* 171.6, 169.6, 155.7, 94.0, 79.6, 69.2, 39.8, 28.4, −12.7.

#### *tert*-Butyl(2-((5-(iodomethyl)isoxazol-3-yl)oxy)ethyl)(methyl)carbamate (16)

This compound was obtained from 14 employing method C. Yellow oil; yield 61%. ^1^H NMR (400 MHz, CDCl_3_) *δ* 5.93 (s, 1H), 4.34 (m, 2H), 4.28 (s, 2H), 3.60 (m, 2H), 2.94 (s, 3H), 1.45 (s, 9H). ^13^C NMR (101 MHz, CDCl_3_) *δ* 171.5, 169.5, 155.5, 94.0, 79.7, 68.1, 47.9, 35.2, 28.4, −12.6.

#### *N*-(2-((5-((3-Fluorophenoxy)methyl)isoxazol-3-yl)oxy)ethyl)-*N*-methylcyclobutanamine hydrochloride (17)

This compound was obtained from 16 employing methods D, E and F. White solid; yield 33%; purity 94.6%. ^1^H NMR (400 MHz, D_2_O) *δ* 7.31 (m, 1H), 6.80 (m, 3H), 6.26 (s, 1H), 5.13 (s, 2H), 4.52 (m, 2H), 3.86–3.71 (m, 1H), 3.70–3.26 (m, 2H), 2.79 (s, 3H), 2.31 (m, 2H), 2.18 (m, 2H), 1.78 (m, 2H). ^13^C NMR (101 MHz, D_2_O) *δ* 171.0, 169.3, 163.3 (d, *J*_C–F_ = 243.7 Hz), 158.4 (d, *J*_C–F_ = 11.0 Hz), 130.9 (d, *J*_C–F_ = 10.1 Hz), 111.0 (d, *J*_C–F_ = 2.9 Hz), 109.0 (d, *J*_C–F_ = 21.3 Hz), 102.9 (d, *J*_C–F_ = 25.4 Hz), 95.51, 63.8, 61.5, 60.0, 52.1, 36.9, 25.9, 12.7. HRMS (ESI): calcd for C_17_H_22_FN_2_O_3_ [M + H]^+^, 321.1609; found, 321.1613.

#### *N*-(2-((5-((4-Fluorophenoxy)methyl)isoxazol-3-yl)oxy)ethyl)-*N*-methylcyclobutanamine hydrochloride (18)

This compound was obtained from 16 employing methods D, E and F. White solid; yield 37%; purity 98.9%. ^1^H NMR (400 MHz, D_2_O) *δ* 7.09 (t, *J* = 7.6 Hz, 2H), 7.02 (s, 2H), 6.32 (s, 1H), 5.14 (s, 2H), 4.59 (m, 2H), 3.93–3.78 (m, 1H), 3.56 (m, 2H), 2.88 (s, 3H), 2.38 (m, 2H), 2.27 (m, 2H), 1.87 (m, 1H). ^13^C NMR (101 MHz, D_2_O) *δ* 171.0, 169.5, 157.8 (d, *J*_C–F_ = 237.5 Hz), 153.3 (d, *J*_C–F_ = 2.0 Hz), 116.7 (d, *J*_C–F_ = 8.4 Hz), 116.1 (d, *J*_C–F_ = 23.5 Hz), 95.5, 63.8, 62.0, 60.0, 52.1, 37.0, 26.1, 25.7, 12.7. HRMS (ESI): calcd for C_17_H_22_FN_2_O_3_ [M + H]^+^, 321.1609; found, 321.1597.

#### *N*-(2-((5-((3-Bromophenoxy)methyl)isoxazol-3-yl)oxy)ethyl)-*N*-methylcyclobutanamine hydrochloride (19)

This compound was obtained from 16 employing methods D, E and F. White solid; yield 36%; purity 98.1%. ^1^H NMR (400 MHz, DMSO-*d*_6_) *δ* 11.49 (br, 1H), 7.28 (d, *J* = 9.2 Hz, 2H), 7.19 (d, *J* = 7.6 Hz, 1H), 7.07 (d, *J* = 8.0 Hz, 1H), 6.48 (s, 1H), 5.25 (s, 2H), 4.59 (m, 2H), 3.76 (m, 1H), 3.58–3.39 (m, 2H), 2.67 (s, 3H), 2.37 (m, 2H), 2.23–2.09 (m, 2H), 1.67 (m, 2H). ^13^C NMR (101 MHz, DMSO-*d*_6_) *δ* 170.6, 168.7, 158.3, 131.3, 124.3, 122.1, 117.6, 114.2, 95.6, 64.2, 60.8, 58.6, 50.6, 36.5, 25.3, 24.9, 12.6. HRMS (ESI): calcd for C_17_H_22_BrN_2_O_3_ [M + H]^+^, 381.0808; found, 381.0800.

#### *N*-(2-((5-((4-Bromophenoxy) methyl) isoxazol-3-yl) oxy) ethyl)-*N*-methyl-cyclobutanamine hydrochloride (20)

This compound was obtained from 16 employing methods D, E and F. White solid; yield 44%; purity 95.9%. ^1^H NMR (400 MHz, DMSO-*d*_6_) *δ* 11.28 (br, 1H), 7.47 (d, *J* = 8.4 Hz, 2H), 7.01 (d, *J* = 8.4 Hz, 2H), 6.45 (s, 1H), 5.19 (s, 2H), 4.65–4.48 (m, 2H), 3.74 (m, 1H), 3.42 (m, 2H), 2.65 (s, 3H), 2.42–2.25 (m, 2H), 2.22–2.08 (m, 2H), 1.67 (m, 2H). ^13^C NMR (101 MHz, DMSO-*d*_6_) *δ* 170.5, 168.7, 156.6, 132.1, 117.0, 112.9, 95.5, 64.1, 60.7, 58.6, 50.6, 36.5, 25.3, 24.8, 12.7. HRMS (ESI): calcd for C_17_H_22_BrN_2_O_3_ [M + H]^+^, 381.0808; found, 381.0789.

#### *N*-(2-((5-((3,4-Difluorophenoxy)methyl)isoxazol-3-yl)oxy)ethyl)-*N*-methylcyclobutanamine hydrochloride (21)

This compound was obtained from 16 employing methods D, E and F. White solid; yield 41%; purity 98.1%. ^1^H NMR (400 MHz, DMSO-*d*_6_) *δ* 11.20 (br, 1H), 7.39 (q, *J* = 9.6 Hz, 1H), 7.28–7.17 (m, 1H), 6.95–6.84 (m, 1H), 6.48 (s, 1H), 5.21 (s, 2H), 4.57 (m, 2H), 3.76 (m, 1H), 3.44 (m, 2H), 2.67 (s, 3H), 2.32 (m, 2H), 2.25–2.02 (m, 2H), 1.70 (m, 2H). ^13^C NMR (101 MHz, DMSO-*d*_6_) *δ* 170.6, 168.6, 153.9 (d, *J*_C–F_ = 9.2 Hz), 149.5 (dd, *J*_C–F_ = 245.3, 13.7 Hz), 144.4 (dd, *J*_C–F_ = 238.6, 12.8 Hz), 117.7 (d, *J*_C–F_ = 18.4 Hz), 111.2 (dd, *J*_C–F_ = 6.1, 3.2 Hz), 104.6 (d, *J*_C–F_ = 20.5 Hz), 95.7, 64.2, 61.3, 58.7, 50.7, 36.6, 25.4, 24.9, 12.6. HRMS (ESI): calcd for C_17_H_21_F_2_N_2_O_3_ [M + H]^+^, 339.1515; found, 339.1497.

#### *N*-(2-((5-((3,5-Difluorophenoxy)methyl)isoxazol-3-yl)oxy)ethyl)-*N*-methylcyclobutanamine hydrochloride (22)

This compound was obtained from 16 employing methods D, E and F. White solid; yield 52%; purity 100%. ^1^H NMR (400 MHz, CDCl_3_) *δ* 6.42 (m, 3H), 5.95 (s, 1H), 4.96 (s, 2H), 4.29 (t, *J* = 6.4 Hz, 2H), 2.89–2.75 (m, 1H), 2.63 (t, *J* = 6.4 Hz, 2H), 2.15 (s, 3H), 2.05–1.93 (m, 2H), 1.91–1.77 (m, 2H), 1.68–1.50 (m, 2H). ^13^C NMR (101 MHz, CDCl_3_) *δ* 171.7, 167.3, 163.6 (dd, *J*_C–F_ = 247.4, 15.5 Hz), 159.5 (t, *J*_C–F_ = 13.6 Hz), 98.7 (dd, *J*_C–F_ = 20.6, 8.4 Hz), 97.4 (t, *J*_C–F_ = 25.8 Hz), 95.5, 67.8, 61.9, 60.5, 52.5, 38.5, 27.7, 13.8. HRMS (ESI): calcd for C_17_H_21_F_2_N_2_O_3_ [M + H]^+^, 339.1515; found, 339.1513.

#### *N*-(2-((5-((3,4-Dichlorophenoxy)methyl)isoxazol-3-yl)oxy)ethyl)-*N*-methylcyclobutanamine hydrochloride (23)

This compound was obtained from 16 employing methods D, E and F. White solid; yield 45%; purity 99.3%. ^1^H NMR (400 MHz, D_2_O) *δ* 7.24 (d, *J* = 6.4 Hz, 1H), 6.95 (s, 1H), 6.81 (d, *J* = 8.0 Hz, 1H), 6.28 (s, 1H), 5.00 (s, 2H), 4.57 (m, 2H), 3.82 (m, 1H), 3.54 (m, 2H), 2.86 (s, 3H), 2.31 (m, 4H), 1.80 (m, 2H). ^13^C NMR (101 MHz, D_2_O) *δ* 170.8, 168.6, 156.5, 132.4, 130.8, 124.6, 116.8, 114.5, 95.7, 63.9, 61.5, 59.9, 52.0, 37.2, 26.0, 12.8. HRMS (ESI): calcd for C_17_H_21_Cl_2_N_2_O_3_ [M + H]^+^, 371.0924; found, 371.0920.

#### *N*-(2-((5-((3, 5-Dichlorophenoxy)methyl)isoxazol-3-yl)oxy)ethyl)-*N*-methylcyclobutanamine hydrochloride (24)

This compound was obtained from 16 employing methods D, E and F. White solid; yield 40%; purity 99.1%. ^1^H NMR (400 MHz, DMSO-*d*_6_) *δ* 11.04 (br, 1H), 7.22 (s, 1H), 7.18 (s, 2H), 6.49 (s, 1H), 5.28 (s, 2H), 4.57 (m, 2H), 3.76 (m, 1H), 3.49 (m, 2H), 2.67 (s, 3H), 2.31 (m, 2H), 2.18 (m, 2H), 1.68 (m, 2H). ^13^C NMR (101 MHz, DMSO-*d*_6_) *δ* 170.6, 168.3, 158.7, 134.7, 121.3, 114.2, 95.9, 64.2, 61.1, 58.7, 50.8, 36.6, 25.4, 25.0, 12.6. HRMS (ESI): calcd for C_17_H_21_Cl_2_N_2_O_3_ [M + H]^+^, 371.0924; found, 371.0923.

#### *N*-(2-((5-((3-Chloro-4-fluorophenoxy)methyl)isoxazol-3-yl)oxy)ethyl)-*N*-methylcyclobutanamine hydrochloride (25)

This compound was obtained from 16 employing methods D, E and F. White solid; yield 37%; purity 97.4%. ^1^H NMR (400 MHz, CDCl_3_) *δ* 7.02 (t, *J* = 8.8 Hz, 1H), 6.95 (dd, *J* = 5.8, 3.0 Hz, 1H), 6.77 (m, 1H), 5.94 (s, 1H), 4.95 (s, 2H), 4.29 (t, *J* = 5.8 Hz, 2H), 2.88–2.77 (m, 1H), 2.64 (t, *J* = 5.8 Hz, 2H), 2.15 (s, 3H), 2.05–1.92 (m, 2H), 1.91–1.75 (m, 2H), 1.62 (m, 2H). ^13^C NMR (101 MHz, DMSO-*d*_6_) *δ* 170.6, 168.6, 154.0 (d, *J*_C–F_ = 2.2 Hz), 152.3 (d, *J*_C–F_ = 239.8 Hz), 119.8 (d, *J*_C–F_ = 19.1 Hz), 117.3 (d, *J*_C–F_ = 22.5 Hz), 116.5, 115.4 (d, *J*_C–F_ = 7.0 Hz), 95.7, 64.2, 61.3, 58.6, 50.6, 36.5, 25.3, 24.8, 12.6. HRMS (ESI): calcd for C_17_H_21_ClFN_2_O_3_ [M + H]^+^, 355.1219; found, 335.1220.

#### *N*-Methyl-*N*-(2-((5-((3-(trifluoromethyl)phenoxy)methyl)isoxazol-3-yl)oxy)ethyl)cyclobutanamine hydrochloride (26)

This compound was obtained from 16 employing methods D, E and F. White solid; yield 46%; purity 96.4%. ^1^H NMR (400 MHz, D_2_O) *δ* 7.19 (d, *J* = 7.2 Hz, 1H), 6.98 (m, 3H), 6.14 (s, 1H), 4.91 (s, 2H), 4.46 (m, 2H), 3.68 (m, 1H), 3.42 (m, 2H), 2.75 (s, 3H), 2.29–2.15 (m, 4H), 1.68 (m, 2H). ^13^C NMR (101 MHz, D_2_O) *δ* 170.8, 168.8, 157.4, 131.2 (q, *J*_C–F_ = 32.2 Hz), 130.4, 123.7 (q, *J*_C–F_ = 272.1 Hz), 118.2 (d, *J*_C–F_ = 3.8 Hz), 117.9, 111.8 (q, *J*_C–F_ = 4.0 Hz), 95.5, 63.8, 61.1, 59.9, 52.0, 37.1, 25.9, 12.6. HRMS (ESI): calcd for C_18_H_22_F_3_N_2_O_3_ [M + H]^+^, 371.1577; found, 371.1565.

#### *N*-Methyl-*N*-(2-((5-((*m*-tolyloxy)methyl)isoxazol-3-yl)oxy)ethyl)cyclobutanamine hydrochloride (27)

This compound was obtained from 16 employing methods D, E and F. White solid; yield 51%. Purity 98.2%. ^1^H NMR (400 MHz, D_2_O) *δ* 7.18 (t, *J* = 7.8 Hz, 1H), 6.84 (d, *J* = 7.2 Hz, 1H), 6.81–6.74 (m, 2H), 6.22 (s, 1H), 5.05 (s, 2H), 4.48 (m, 2H), 3.77 (m, 1H), 3.46 (m, 2H), 2.77 (s, 3H), 2.29 (m, 2H), 2.21 (s, 3H), 2.17 (m, 2H), 1.76 (m, 2H). ^13^C NMR (101 MHz, D_2_O) *δ* 171.0, 169.7, 157.2, 140.5, 129.7, 123.1, 115.8, 112.0, 95.4, 63.7, 61.2, 60.0, 52.1, 36.9, 25.9, 20.5, 12.7. HRMS (ESI): calcd for C_18_H_25_N_2_O_3_ [M + H]^+^, 317.1860; found, 317.1866.

#### *N*-(2-((5-(Phenoxymethyl)isoxazol-3-yl)oxy)ethyl)cyclobutanamine hydrochloride (28)

This compound was obtained from 15 employing methods D, E and F. White solid; yield 27%; purity 94.6%. ^1^H NMR (400 MHz, CDCl_3_) *δ* 7.30 (t, *J* = 8.0 Hz, 2H), 7.00 (t, *J* = 7.4 Hz, 1H), 6.94 (d, *J* = 8.0 Hz, 2H), 5.98 (s, 1H), 5.04 (s, 2H), 4.33 (t, *J* = 5.2 Hz, 2H), 3.44–3.26 (m, 1H), 2.96 (t, *J* = 5.2 Hz, 2H), 2.54 (br, 1H), 2.32–2.16 (m, 2H), 1.88–1.51 (m, 4H). ^13^C NMR (101 MHz, CDCl_3_) *δ* 171.7, 168.9, 157.7, 129.6, 121.9, 114.8, 94.9, 69.2, 61.6, 53.9, 45.6, 30.7, 14.7. HRMS (ESI): calcd for C_16_H_21_N_2_O_3_ [M + H]^+^, 289.1560; found, 289.1547.

#### *N*-(2-((5-((3-Chlorophenoxy)methyl)isoxazol-3-yl)oxy)ethyl)cyclobutanamine hydrochloride (29)

This compound was obtained from 15 employing methods D, E and F. White solid; yield 55%; purity 96.7%. ^1^H NMR (400 MHz, CDCl_3_) *δ* 7.19 (t, *J* = 8.2 Hz, 1H), 6.97 (d, *J* = 8.0 Hz, 1H), 6.93 (s, 1H), 6.81 (dd, *J* = 8.2, 1.8 Hz, 1H), 5.96 (s, 1H), 4.99 (s, 2H), 4.29 (t, *J* = 4.0 Hz, 2H), 3.34–3.21 (m, 1H), 2.91 (t, *J* = 4.0 Hz, 2H), 2.26–2.12 (m, 2H), 1.75–1.57 (m, 4H), 1.51 (m, 1H). ^13^C NMR (101 MHz, CDCl_3_) *δ* 171.7, 168.1, 158.4, 135.1, 130.4, 122.1, 115.4, 113.1, 95.1, 69.8, 61.7, 54.0, 45.7, 31.2, 14.7. HRMS (ESI): calcd for C_16_H_20_ClN_2_O_3_ [M + H]^+^, 323.1157; found, 323.1171.

#### *N*-Cyclobutyl-*N*-(2-((5-(phenoxymethyl)isoxazol-3-yl)oxy)ethyl)cyclobutanamine hydrochloride (30)

This compound was obtained from 15 employing methods D, E and F. White solid yield 24%; purity 98.4%. ^1^H NMR (400 MHz, CDCl_3_) *δ* 7.29 (t, *J* = 8.0 Hz, 2H), 6.99 (t, *J* = 7.4 Hz, 1H), 6.94 (d, *J* = 8.0 Hz, 2H), 5.96 (s, 1H), 5.02 (s, 2H), 4.25 (t, *J* = 6.2 Hz, 2H), 3.28–3.14 (m, 2H), 2.89 (t, *J* = 6.2 Hz, 2H), 2.11–1.85 (m, 8H), 1.72–1.50 (m, 4H). ^13^C NMR (101 MHz, CDCl_3_) *δ* 171.6, 168.8, 157.7, 129.6, 121.8, 114.8, 94.9, 67.8, 61.6, 57.4, 46.2, 28.8, 15.1. HRMS (ESI): calcd for C_20_H_27_N_2_O_3_ [M + H]^+^, 343.2016; found, 343.2025.

### General procedures for vitro binding studies

Radioligand competition studies were carried out by the National Institute of Mental Health's Psychoactive Drug Screening Program, Contract #HHSN-271-2008-00025-C (NIMH PDSP). The general procedures are listed as follows:

All the test compounds solution (1 mg mL^−1^) were prepared by dissolving in DMSO or buffer solvent (50 mM Tris–HCl, pH = 8.0). The filter-mats were presoaked in 0.5% aqueous polyethyleneimine solution for 2 h at rt before use. All binding experiments were carried out in poly-l-lysine-coated 96-well plate and the final test concentration (50 μM, 5 μM, 1.5 μM, 500 nM, 150 nM, 50 nM, 15 nM, 5 nM, 1.5 nM, 0.5 nM, 0.05 nM) were prepared by addition of Tris–HCl solution (50 mM, pH = 8.0). The following specific radioligands and tissue source were used: (a) σ1 receptor, [^3^H]-(+)-pentazocine, Guinea pig brain homogenate; (b) σ2 receptor, [^3^H]-DTG, PC-12 cells. 100 μL of corresponding radioligand solution, 100 μL test compound solution and 50 μL of the respective receptor preparation into each well of the plate (total volume 250 μL). During the incubation, the plates were shaken at a speed of 500–600 rpm at the specified temperature (σ1 receptor, 37 °C, 150 min; σ2 receptor, rt, 120 min). The incubation was followed by a rapid vacuum filtration through Whatman GF/B glass filters, and the filtrates were washed twice with 10 mL of cold buffer and transferred to the 6 mL scintillation vials. EcoScint scintillation fluid (4.0 mL) was added, and the radioactivity bound was measured using a Beckman LS 6500 liquid scintillation counter. All experiments were performed at least three times.

For experimental details please refer to the PDSP website http://pdsp.med.unc.edu/.^28^

### General procedures for cellular evaluation

#### NGF-induced neurite outgrowth in PC-12 cells

PC-12 cells, from rat pheochromocytoma cell lines, were obtained from the American Type Culture Collection (ATCC, Manassas, VA, USA). Undifferentiated PC12 cells were cultured in DMEM complete medium with 10% heat-inactivated horse serum, 5% fetal bovine serum, 100 U mL^−1^ penicillin, and 100 μg mL^−1^ streptomycin and maintained at 37 °C in a humidified incubator supplemented with 5% CO_2_. To induce differentiation, PC12 cells were seeded at a density of 1 × 10^4^ cells per mL on a poly-l-lysine-coated 96-well plate and cultured in complete medium containing 2.5 ng mL^−1^ NGF or different tested compounds (10 μM) concomitant with 2.5 ng mL^−1^ NGF administration. Four days after incubation, morphometric analysis was performed on digitized images of live cells taken under phase contrast illumination with an inverted microscope linked to a camera. Images of five fields per well were taken with an average of 20 cells per field. The number of differentiated cells was determined by counting cells that had at least one neurite with a length equal to the cell body diameter and was expressed as a percentage of the total cells in the field. The counting was performed in a blinded manner. All experiments were performed at least five times.

#### Neurite outgrowth in N1E-115 cells

N1E-115 cells, the mouse neuroblastoma cell lines, were obtained from the American Type Culture Collection (ATCC, Manassas, VA, USA). Undifferentiated N1E115 cells were cultured in DMEM complete medium with 10% fetal bovine serum, 100 U mL^−1^ of penicillin, and 100 μg mL^−1^ of streptomycin and maintained at 37 °C in a humidified incubator supplemented with 5% CO_2_. N1E-115 cells were seeded at a density of 1 × 10^4^ cells per mL on poly-l-lysine-coated 96-well plates and grown with DMSO or different tested compounds (10 μM) in DMSO solution for four days. Four days after incubation, morphometric analysis was performed on digitized images of live cells taken under phase contrast illumination with an inverted microscope linked to a camera. Images of five fields per well were taken with an average of 100 cells per field. Cells that had at least one neurite with a length that was twice as long as the body diameter were expressed as a percentage of the total cells in the field. The counting was performed in a blinded manner. All experiments were performed at least five times.

#### Positive control drugs

Compounds RC-33 (5) and compound 7 were synthesized according to procedures described in literatures.^23,24,27^

##### RC-33(5)

White solid; ^1^H NMR (400 MHz, DMSO-*d*_6_) *δ* 10.36 (s, 1H), 7.64 (m, 4H), 7.46 (t, *J* = 7.6 Hz, 2H), 7.36 (d, *J* = 8.1 Hz, 3H), 3.03 (m, 2H), 2.88–2.68 (m, 4H), 2.06 (m, 2H), 1.72 (m, 6H), 1.43–1.28 (m, 1H), 1.25 (d, *J* = 6.9 Hz, 3H). ^13^C NMR (101 MHz, DMSO-*d*_6_) *δ* 144.9, 139.9, 138.2, 128.9, 127.4, 127.2, 126.8, 126.5, 54.7, 51.8, 51.8, 36.7, 30.7, 22.3, 21.4.

##### Compound 7

White solid; ^1^H NMR (400 MHz, DMSO-*d*_6_) *δ* 10.99 (s, 1H), 7.36–7.28 (m, 2H), 7.03 (d, *J* = 8.0 Hz, 2H), 6.99 (t, *J* = 8.0 Hz, 1H), 6.45 (s, 1H), 5.19 (s, 2H), 4.56 (m, 2H), 3.81–3.68 (m, 1H), 2.67 (s, 3H), 2.38–2.24 (m, 2H), 2.17 (m, 2H), 1.68 (m, 2H). ^13^C NMR (101 MHz, DMSO-*d*_6_) *δ* 170.9, 169.6, 157.0, 129.9, 122.3, 115.0, 95.4, 63.6, 61.1, 59.9, 52.0, 36.8, 25.9, 12.6.

## Conflicts of interest

There are no conflicts of interest to declare.

## Abbreviations

AD: Alzheimer's disease
PD: Parkinson's disease
ALS: Amyotrophic lateral sclerosis
HD: Huntington's disease
CNS: Central nervous system
ER: Endoplasmic reticulum
NMDA: *N*-Methyl-d-aspartate
IP_3_: Inositol 1,4,5-trisphosphate
NGF: Nerve growth factor
SAR: Structure–activity relationship
NIMH-PDSP: National Institute of Mental Health-Psychoactive Drug Screening Program
CNS MPO: Central nervous system multiparameter optimization
DAT: Dopamine transporter
NET: Norepinephrine transporter
SERT: Serotonin transporter
CC: Column chromatography
rt: Room temperature

## Supplementary Material

RA-008-C8RA00072G-s001
