# Supplementary material for: Discovery of N-cyclobutylaminoethoxyisoxazole derivatives as novel sigma-1 receptor ligands with neurite outgrowth efficacy in cells
Source: RSC Adv. 2018 Feb 14;8(13):7080–8. doi: 10.1039/c8ra00072g (PMC9078399; doi:10.1039/c8ra00072g)

Electronic Supplementary Material (ESI) for RSC Advances.
This journal is © The Royal Society of Chemistry 2018

Supplementary Information

Discovery of N-Cyclobutylaminoethoxyisoxazole Derivatives as Novel Sigma-1 Receptor Ligands with Neurite Outgrowth Efficacy in Cells

Hao Sun [a, +], Yun-Jie Wang [b, +], Wen-Wen Shi [a, +], Fan Yang [a], Jie Tang [a], Tao Pang [b, *], Li-Fang Yu [a, *].

*Corresponding authors

[a]Shanghai Engineering Research Center of Molecular Therapeutics and New Drug Development, School of Chemistry and Molecular Engineering, East China Normal University, 3663 North Zhongshan Road, Shanghai 200062, China

E-mail: lfyu@sat.ecnu.edu.cn; Tel.: +86-21-62231385

[b]Jiangsu Key Laboratory of Drug Screening, State Key Laboratory of Natural Medicines, China Pharmaceutical University, Nanjing 210009, PR China

E-mail: tpang@cpu.edu.cn; Tel.: +86-25-83271043

[+]These authors contributed equally

Contents

^{1}H and ^{13}C NMR spectra of all final compounds

^{1}H NMR and ^{13}C NMR spectra of compound **17**

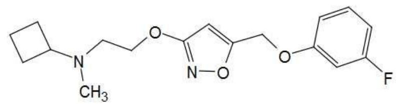

^{1}H NMR (400 MHz, D_2O) δ 7.31 (m, 1H), 6.80 (m, 3H), 6.26 (s, 1H), 5.13 (s, 2H), 4.52 (m, 2H), 3.86 – 3.71 (m, 1H), 3.70 – 3.26 (m, 2H), 2.79 (s, 3H), 2.31 (m, 2H), 2.18 (m, 2H), 1.78 (m, 2H).

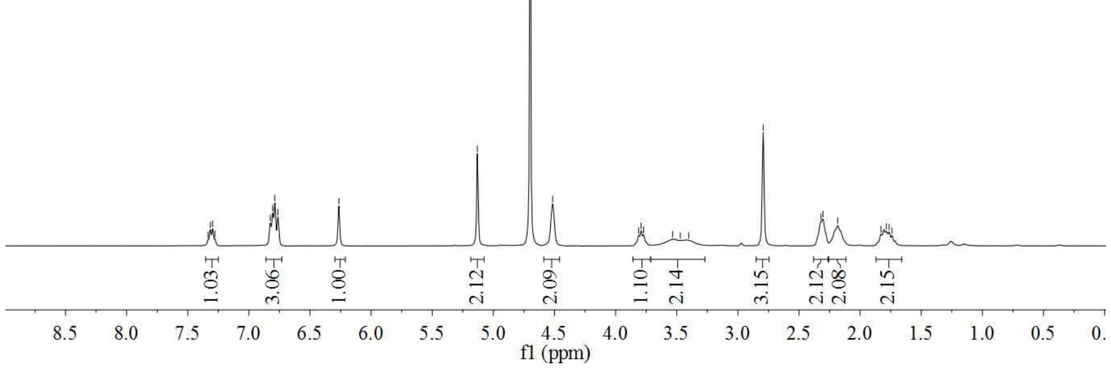

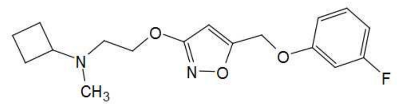

^{13}C NMR (101 MHz, D_2O) δ 171.0, 169.3, 163.3 (d, J = 243.7 Hz), 158.4 (d, J = 11.0 Hz), 130.9 (d, J = 10.1 Hz), 111.0 (d, J = 2.9 Hz), 109.0 (d, J = 21.3 Hz), 102.9 (d, J = 25.4 Hz), 95.51 , 63.8, 61.5, 60.0, 52.1, 36.9, 25.9, 12.7.

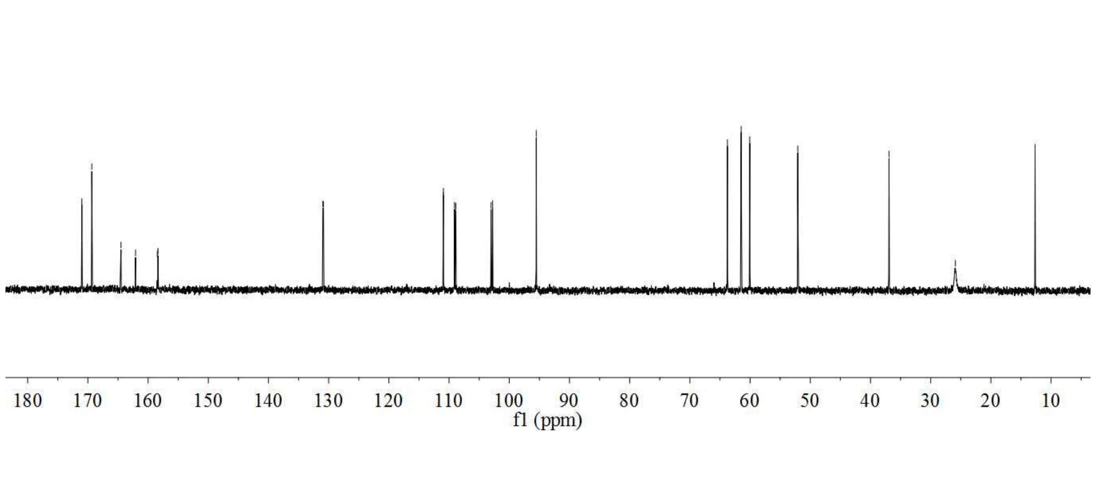

^{1}H NMR and ^{13}C NMR spectra of compound **18**

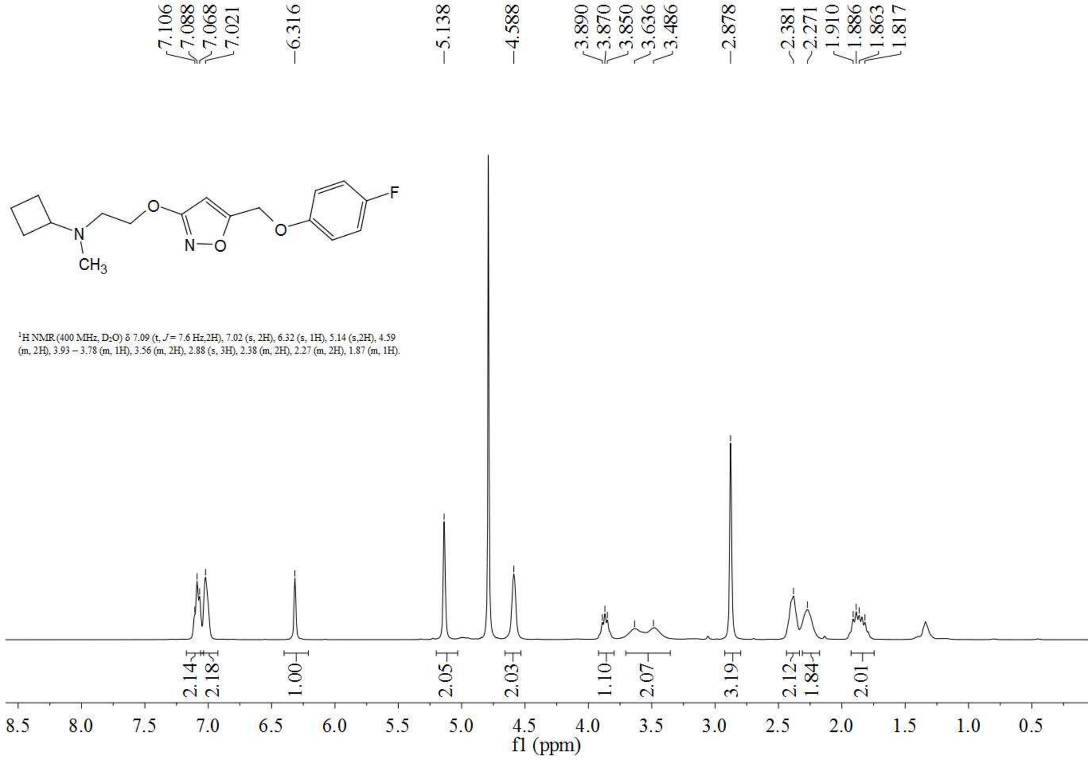

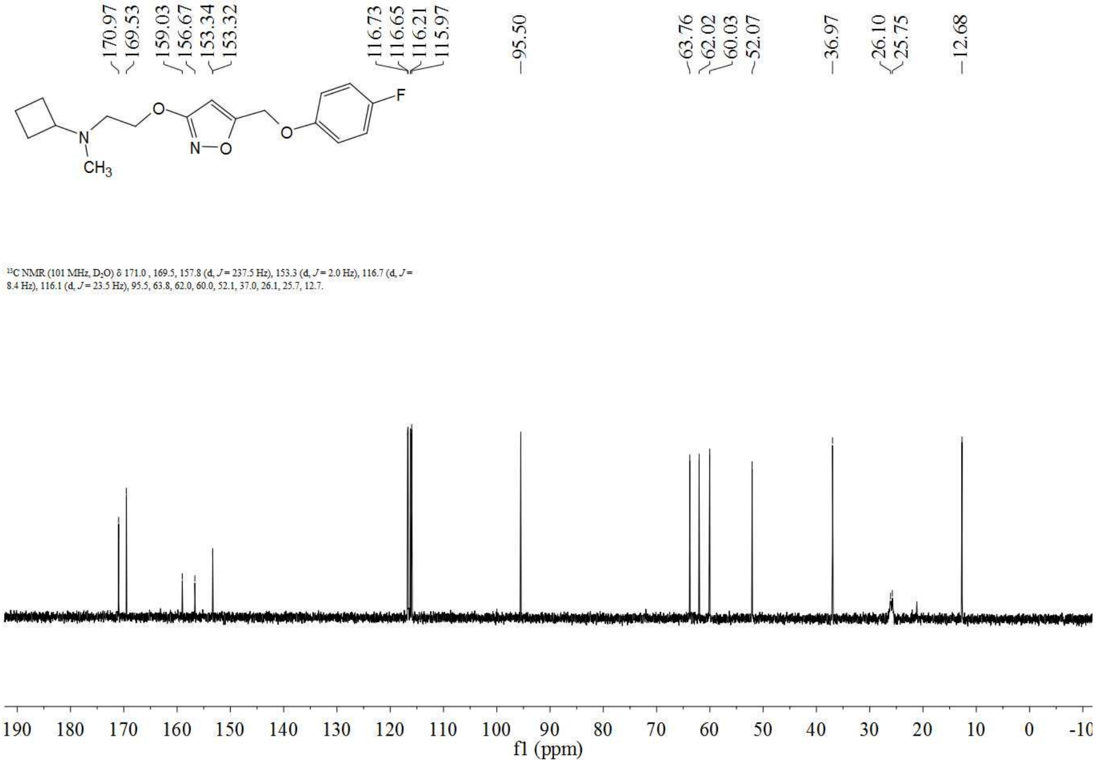

^{1}H NMR and ^{13}C NMR spectra of compound **19**

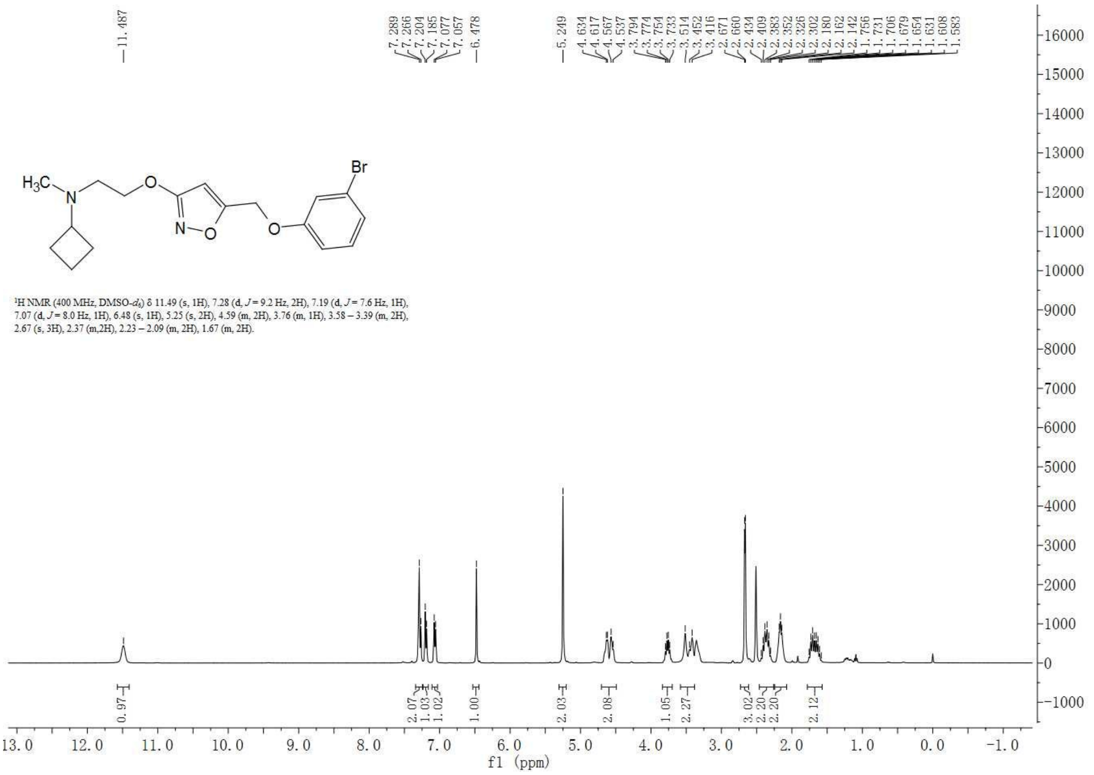

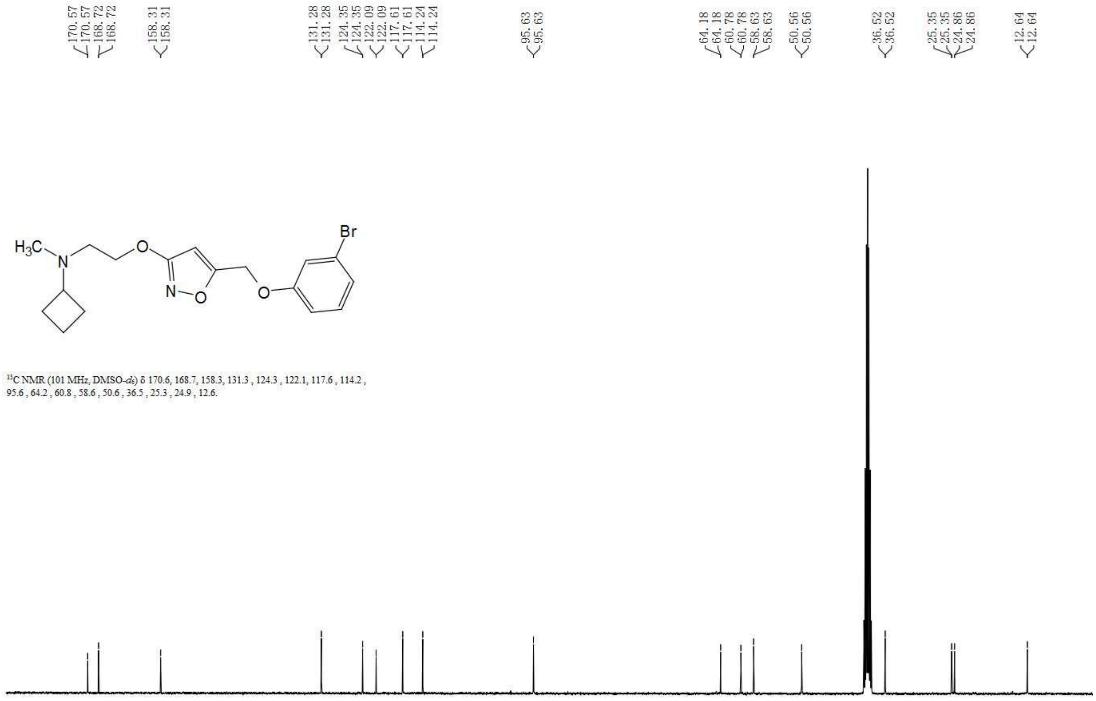

^{1}H NMR and ^{13}C NMR spectra of compound **20**

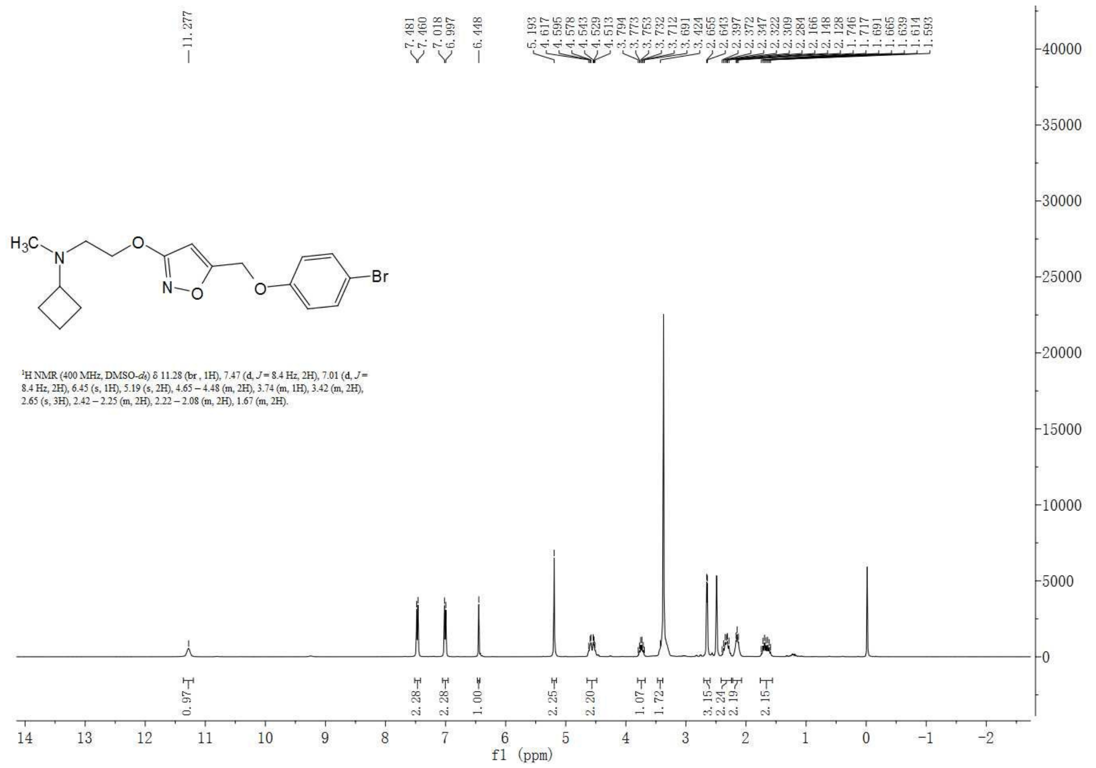

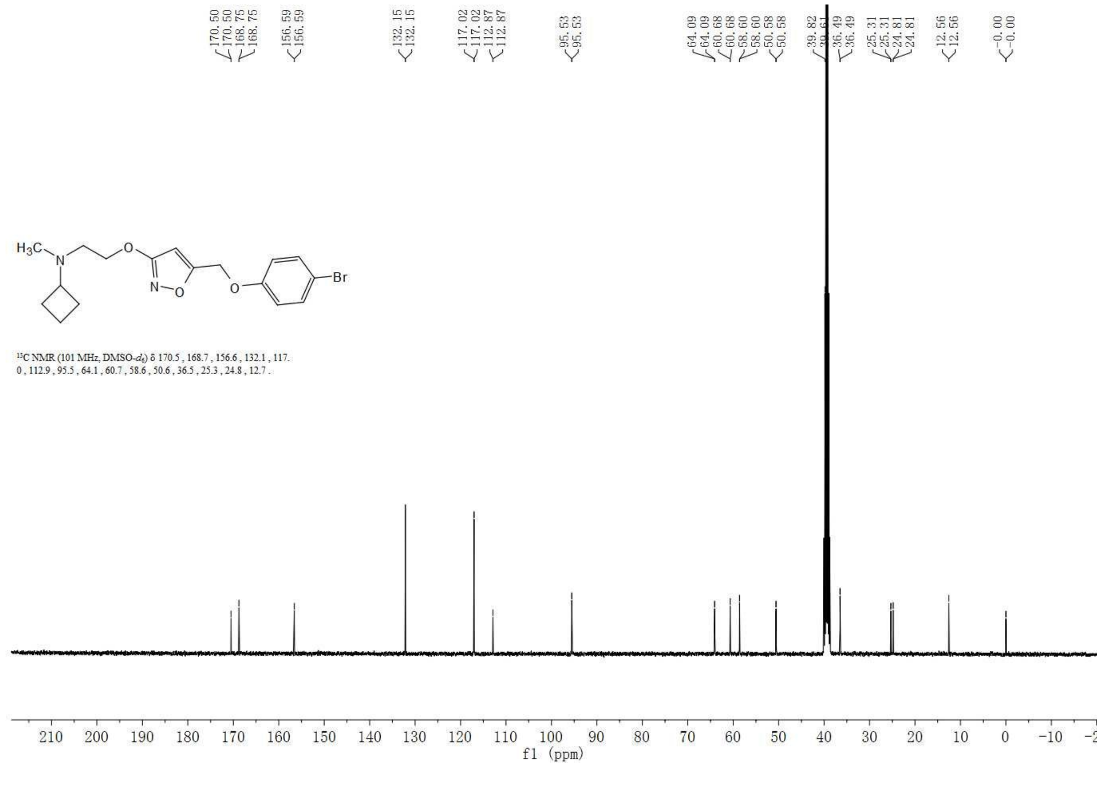

^{1}H NMR and ^{13}C NMR spectra of compound **21**

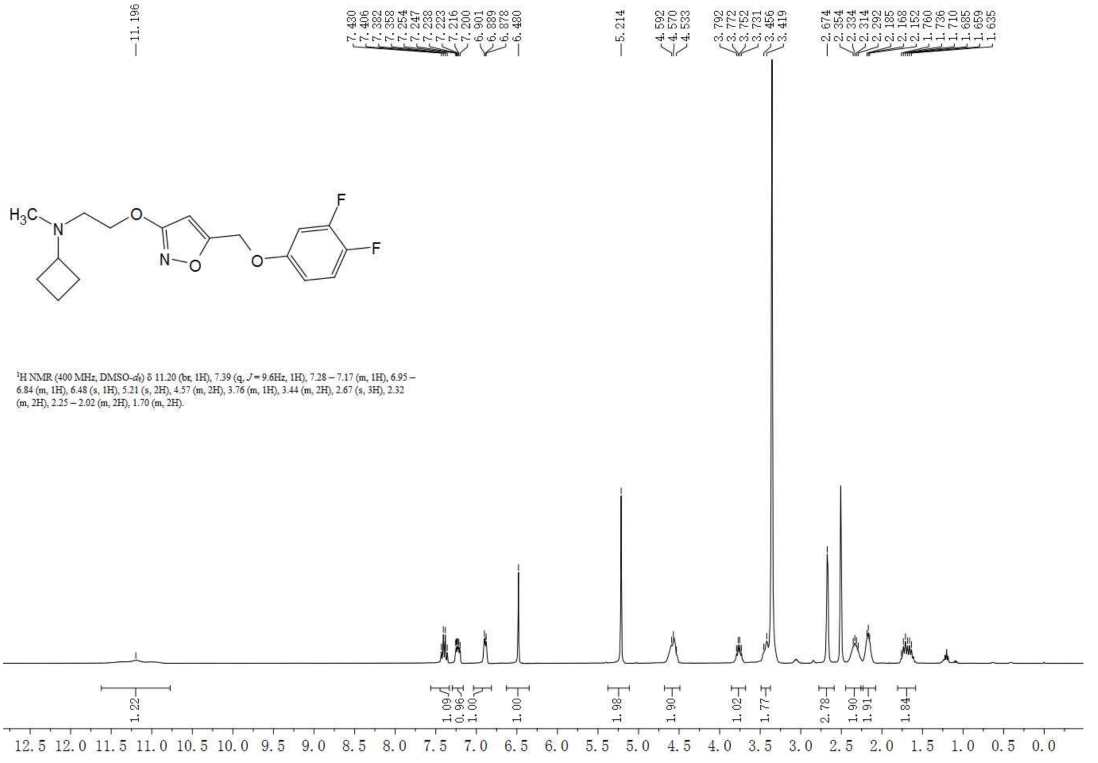

^{1}H NMR (400 MHz, DMSO-d_6) δ 11.20 (br, 1H), 7.39 (q, J = 9.6Hz, 1H), 7.28 – 7.17 (m, 1H), 6.95 – 6.84 (m, 1H), 6.48 (s, 1H), 5.21 (s, 2H), 4.57 (m, 2H), 3.76 (m, 1H), 3.44 (m, 2H), 2.67 (s, 3H), 2.32 (m, 2H), 2.25 – 2.02 (m, 2H), 1.70 (m, 2H).

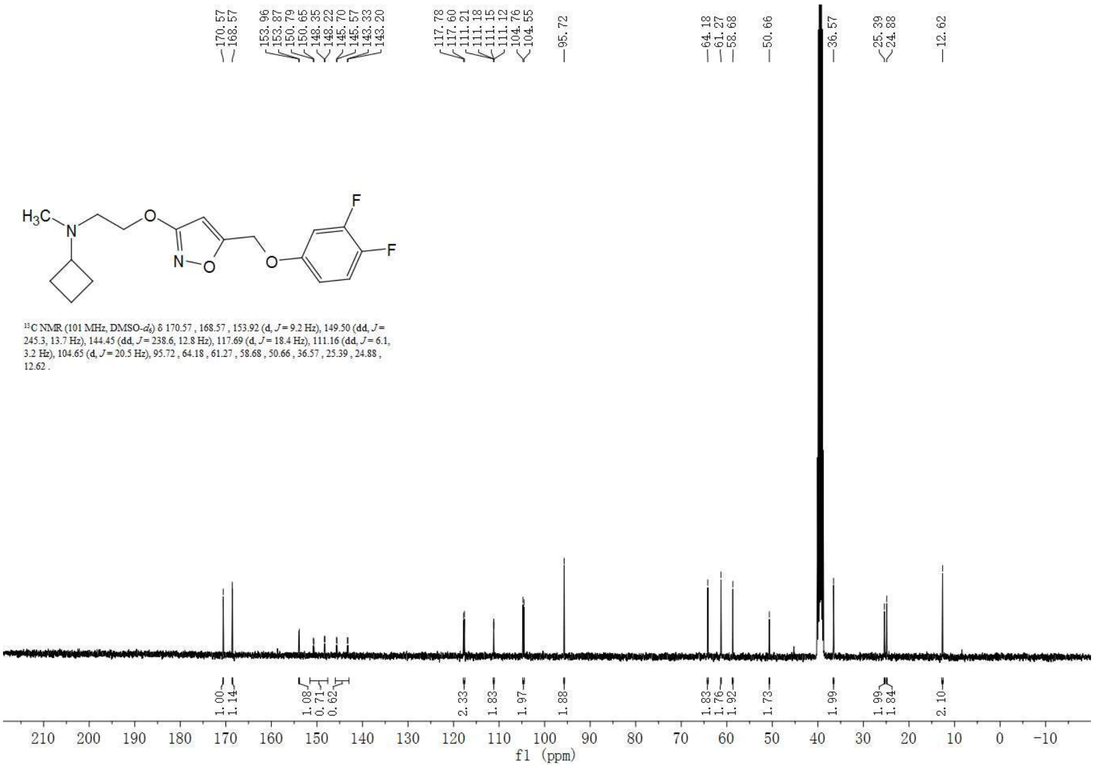

^{13}C NMR (101 MHz, DMSO-d_6) δ 170.57 , 168.57 , 153.92 (d, J = 9.2 Hz), 149.50 (dd, J = 245.3, 13.7 Hz), 144.45 (dd, J = 238.6, 12.8 Hz), 117.69 (d, J = 18.4 Hz), 111.16 (dd, J = 6.1, 3.2 Hz), 104.65 (d, J = 20.5 Hz), 95.72 , 64.18 , 61.27 , 58.68 , 50.66 , 36.57 , 25.39 , 24.88 , 12.62 .

^{1}H NMR and ^{13}C NMR spectra of compound **22**

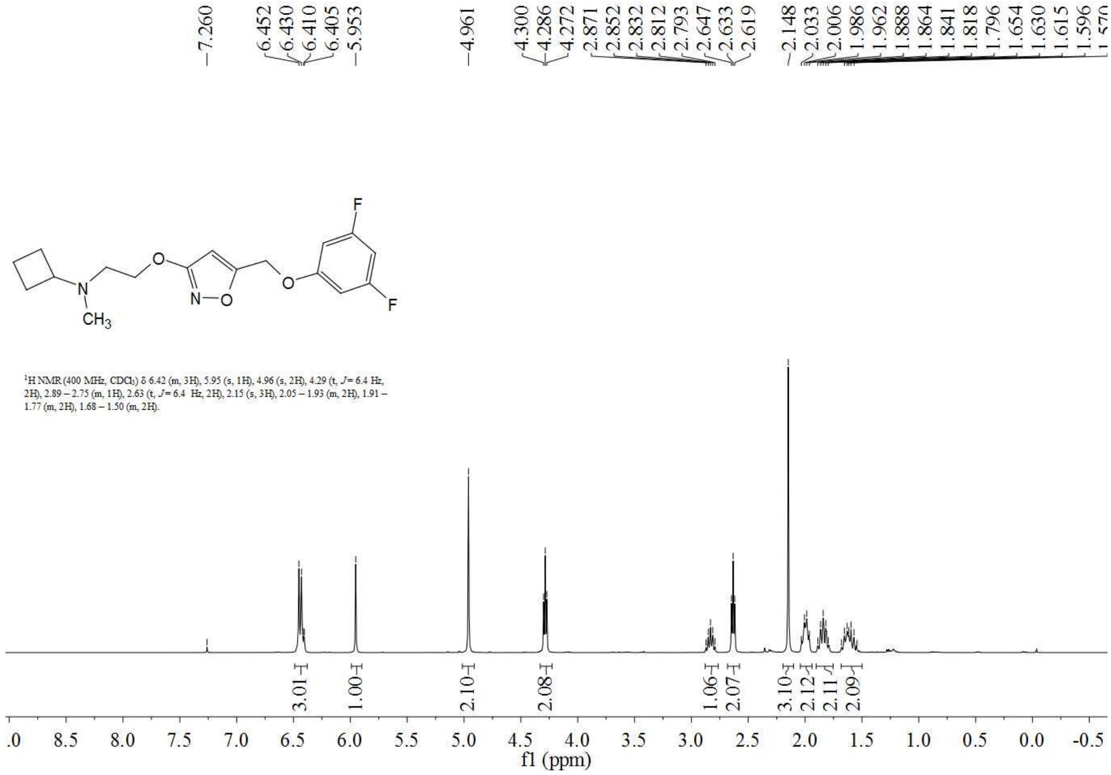

^{1}H NMR (400 MHz, $CDCl_3$) δ 6.42 (m, 3H), 5.95 (s, 1H), 4.96 (s, 2H), 4.29 (t, J= 6.4 Hz, 2H), 2.89 – 2.75 (m, 1H), 2.63 (t, J= 6.4 Hz, 2H), 2.15 (s, 3H), 2.05 – 1.93 (m, 2H), 1.91 – 1.77 (m, 2H), 1.68 – 1.50 (m, 2H).

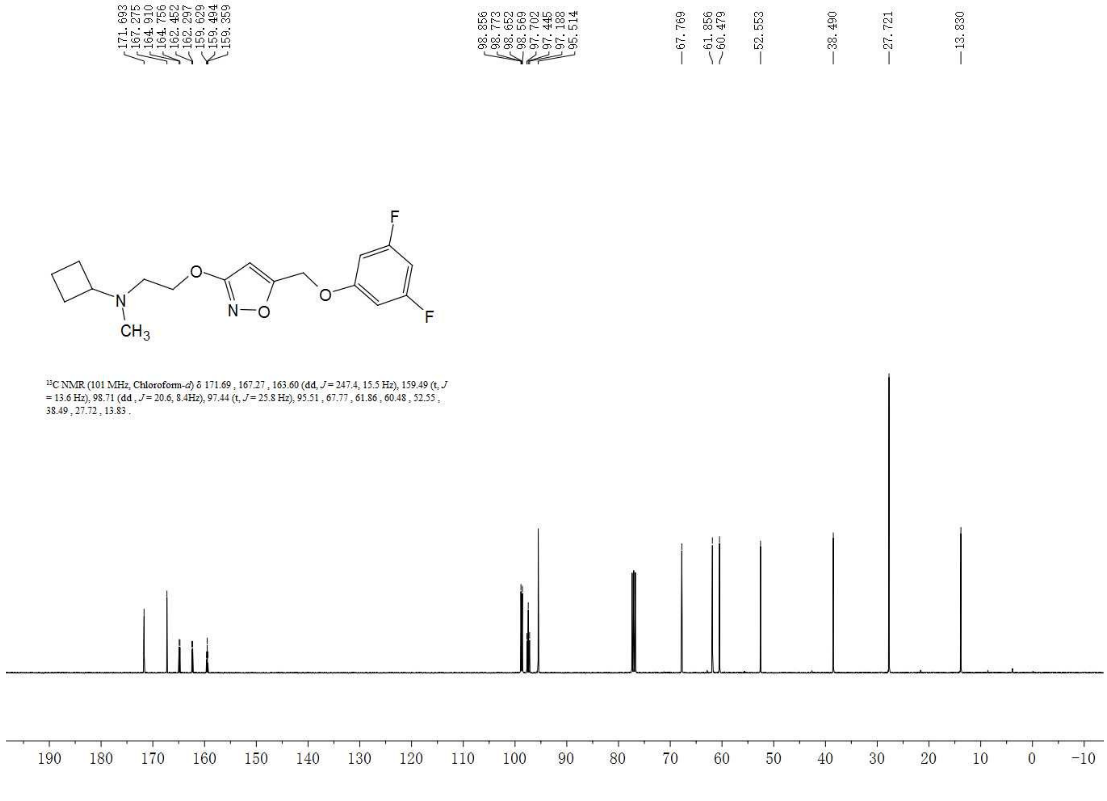

^{13}C NMR (101 MHz, Chloroform-d) δ 171.69 , 167.27 , 163.60 (dd, J = 247.4, 15.5 Hz), 159.49 (t, J = 13.6 Hz), 98.71 (dd , J = 20.6, 8.4Hz), 97.44 (t, J = 25.8 Hz), 95.51 , 67.77 , 61.86 , 60.48 , 52.55 , 38.49 , 27.72 , 13.83 .

^{1}H NMR and ^{13}C NMR spectra of compound **23**

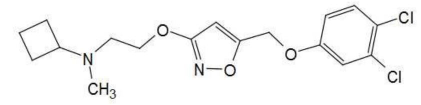

^{1}H NMR (400 MHz, D_2O) δ 7.24 (d, J = 6.4Hz, 1H), 6.95 (s, 1H), 6.81 (d, J = 8.0 Hz, 1H), 6.28 (s, 1H), 5.00 (s, 2H), 4.57 (m, 2H), 3.82 (m, 1H), 3.54 (m , 2H), 2.86 (s, 3H), 2.31 (m, 4H), 1.80 (m, 2H).

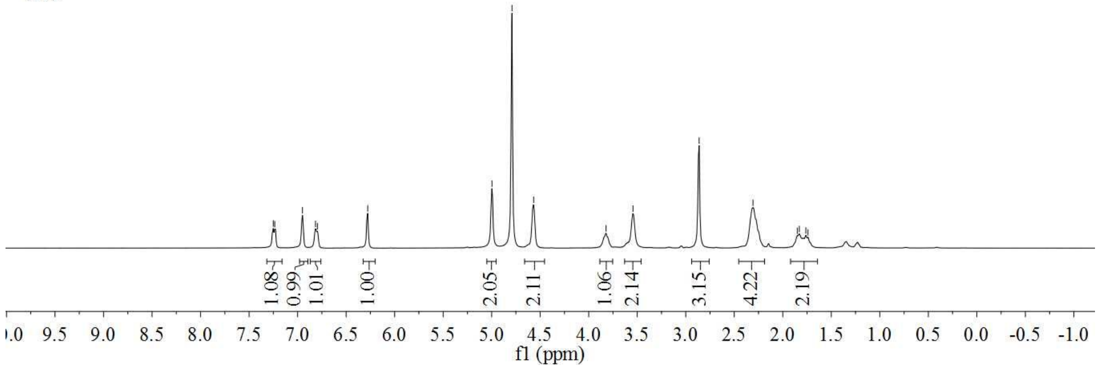

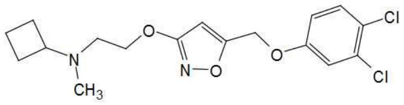

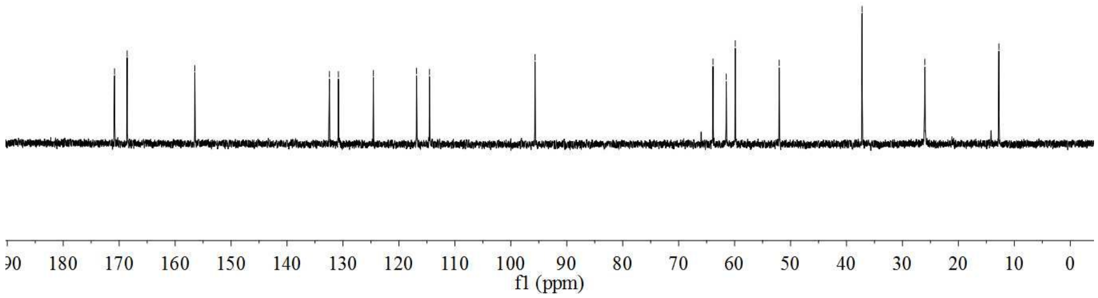

^{1}H NMR and ^{13}C NMR spectra of compound **24**

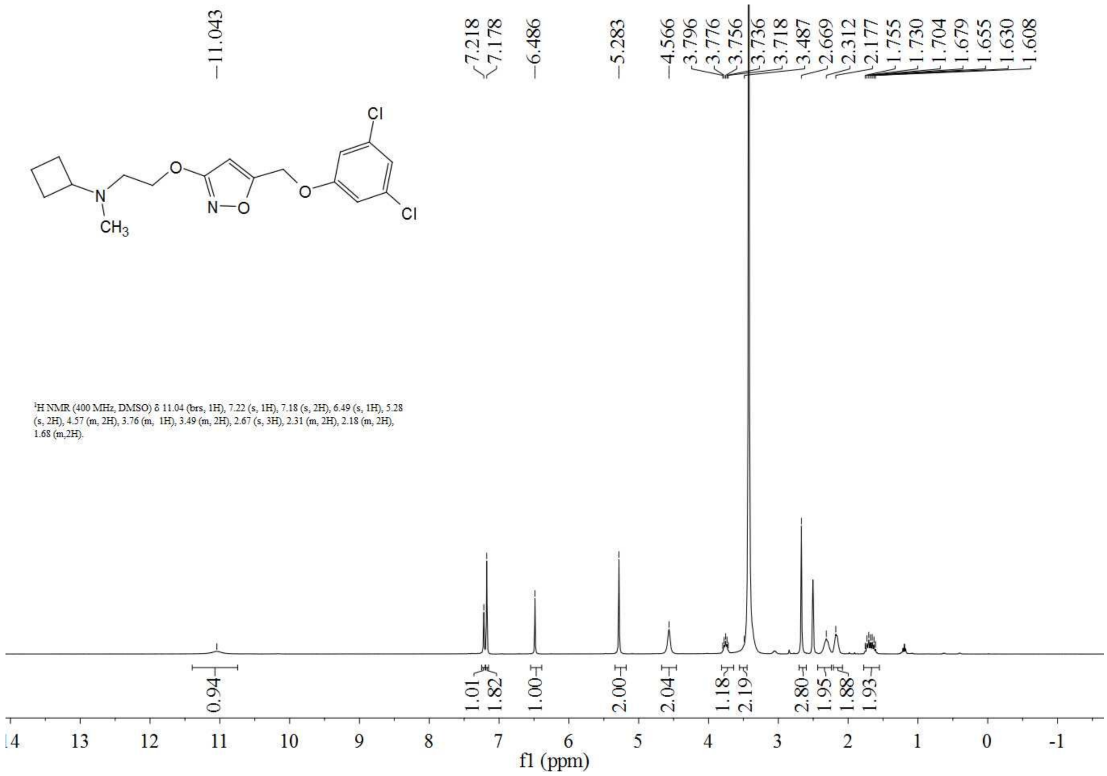

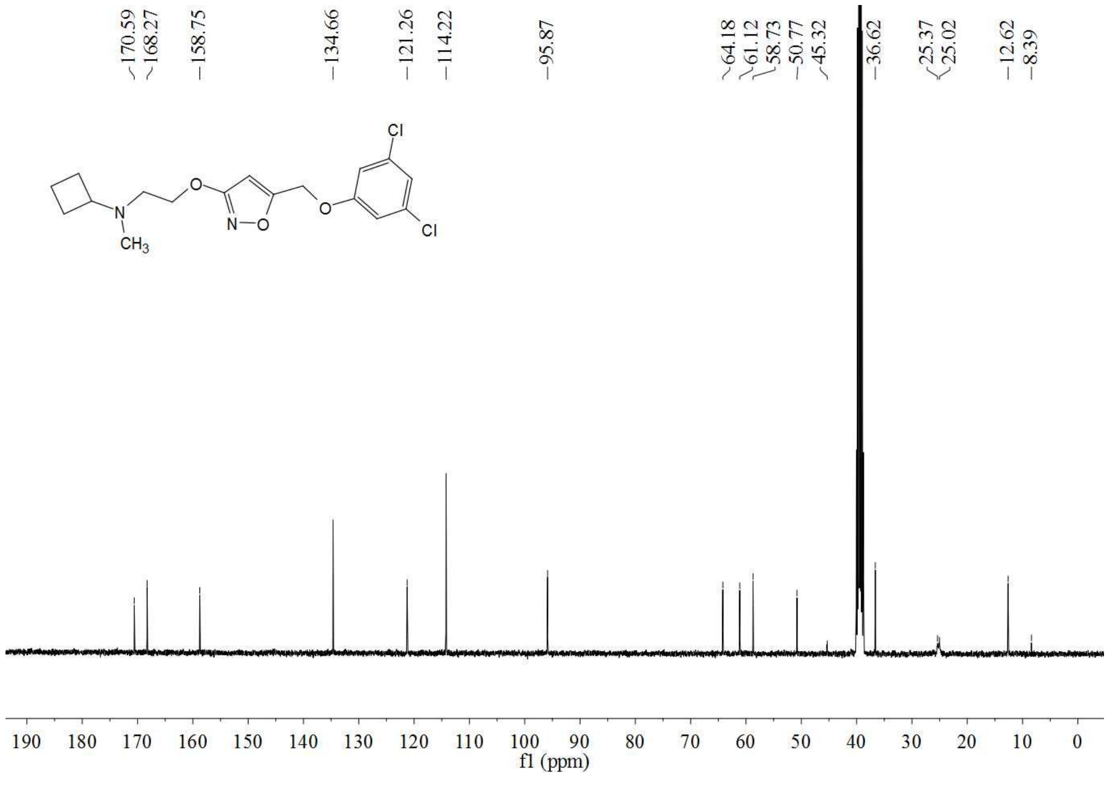

^{1}H NMR and ^{13}C NMR spectra of compound **25**

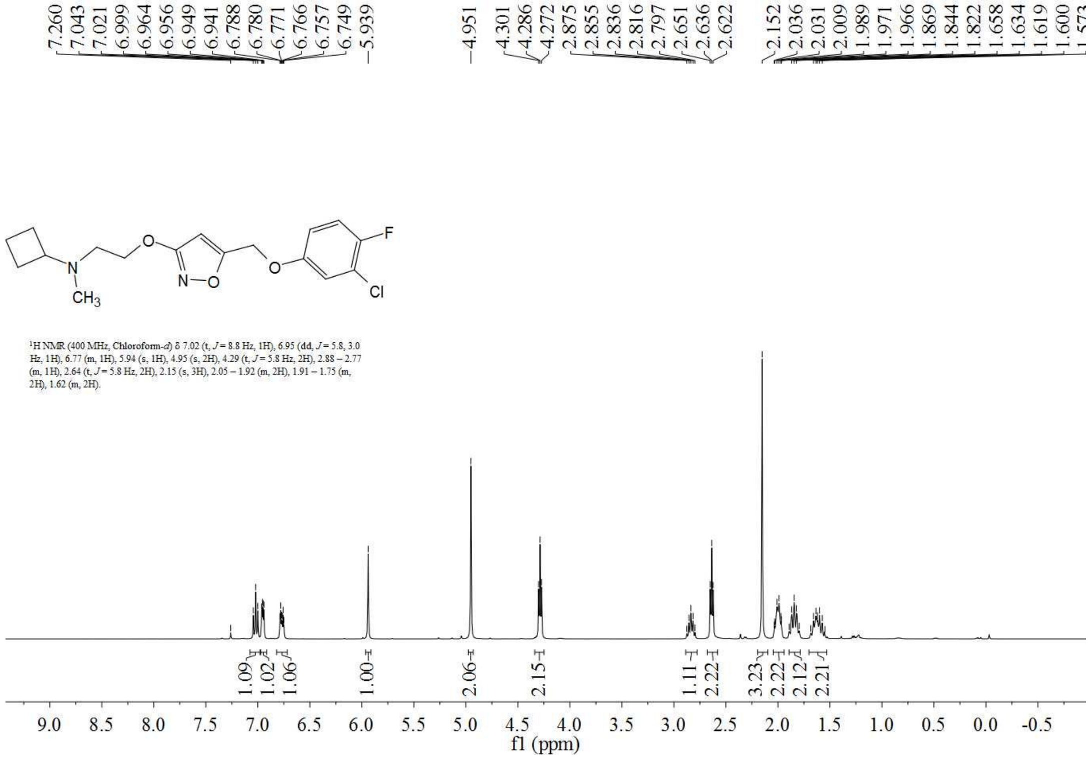

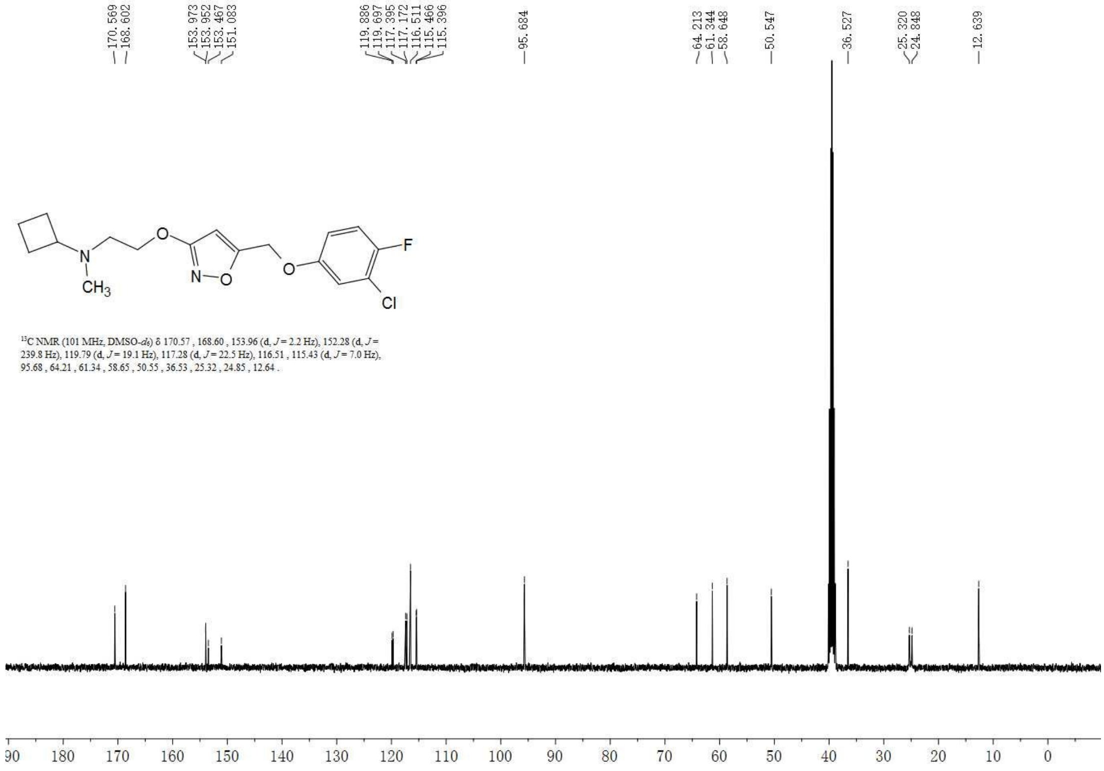

^{1}H NMR and ^{13}C NMR spectra of compound **26**

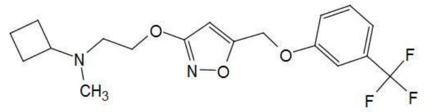

^{1}H NMR (400 MHz, D_2O) δ 7.19 (d, J = 7.2 Hz, 1H), 6.98 (m, 3H), 6.14 (s, 1H), 4.91 (s, 2H), 4.46 (m, 2H), 3.68 (m, 1H), 3.42 (m, 2H), 2.75 (s, 3H), 2.29 – 2.15 (m, 4H), 1.68 (m, 2H).

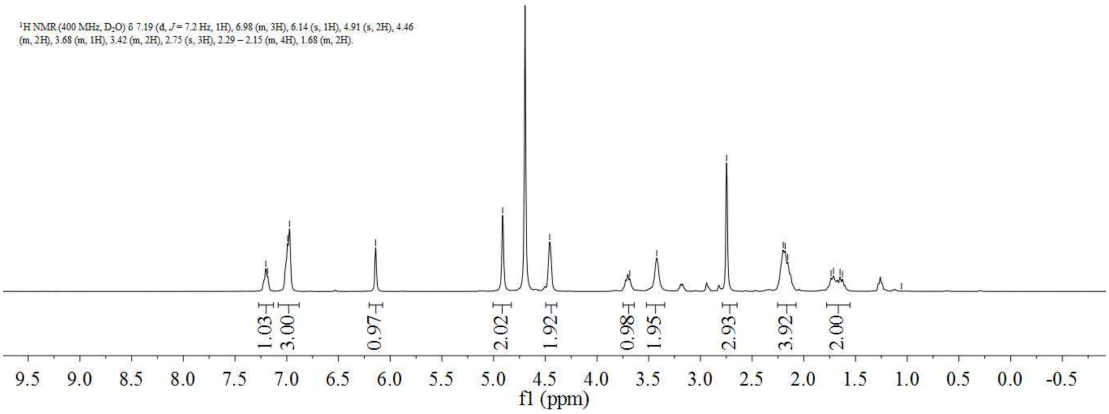

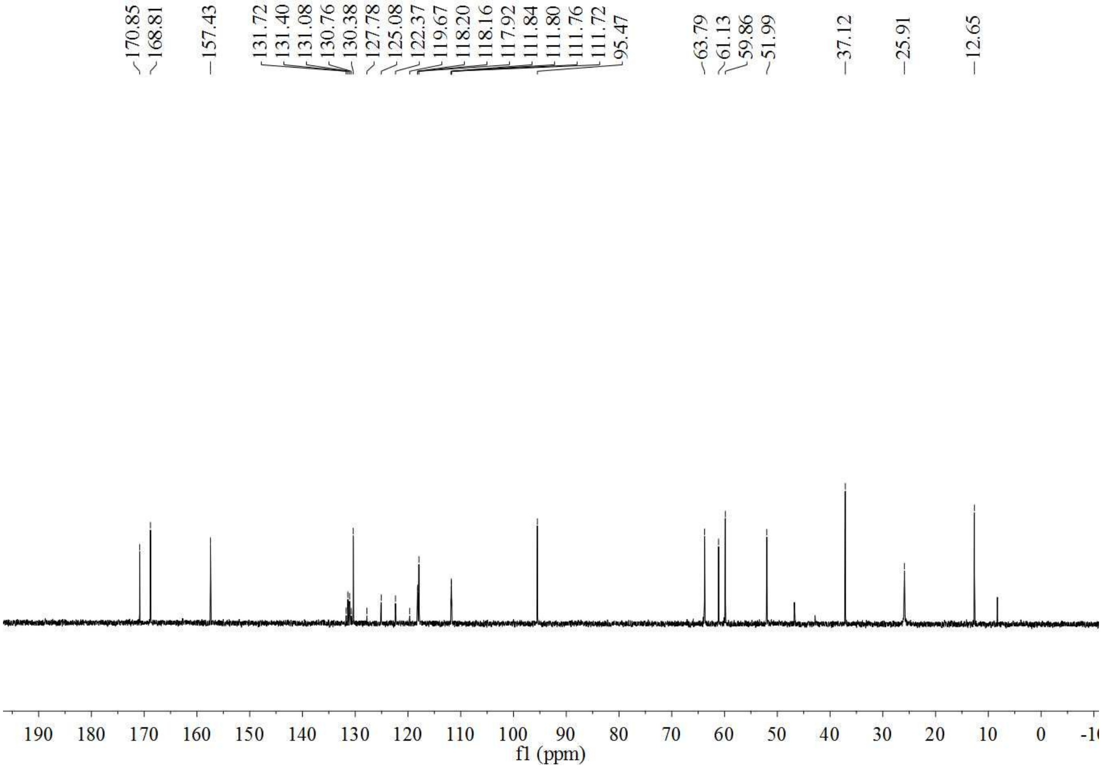

^{1}H NMR and ^{13}C NMR spectra of compound **27**

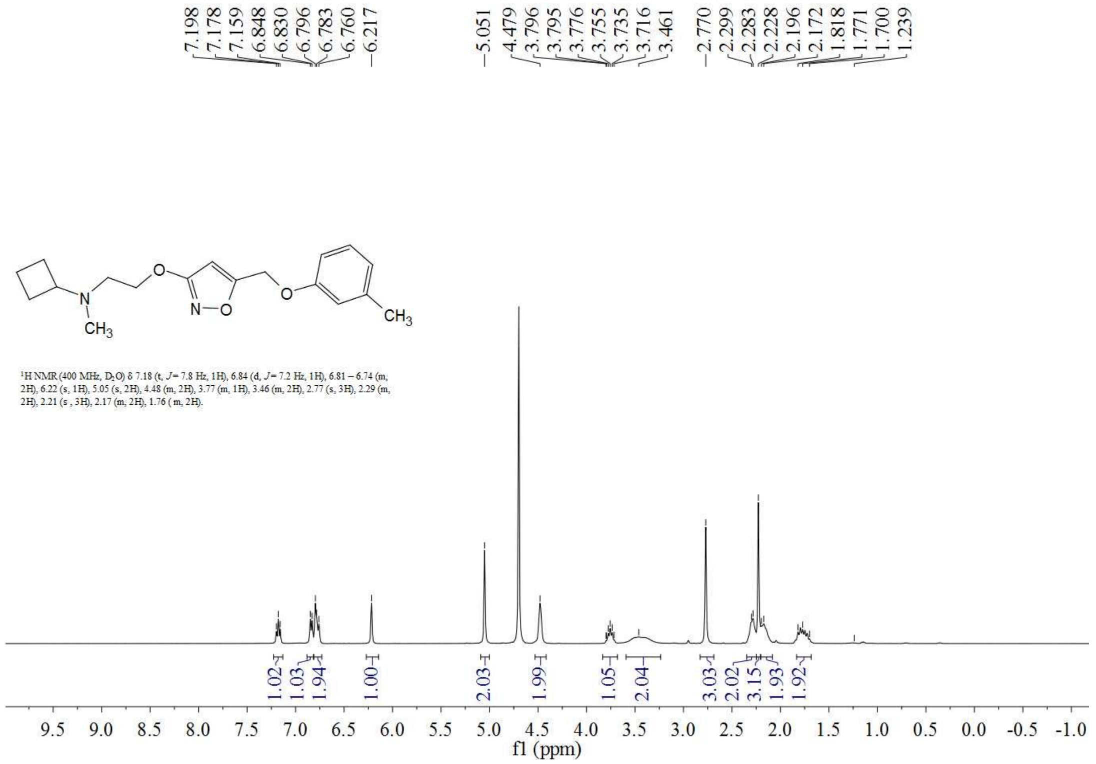

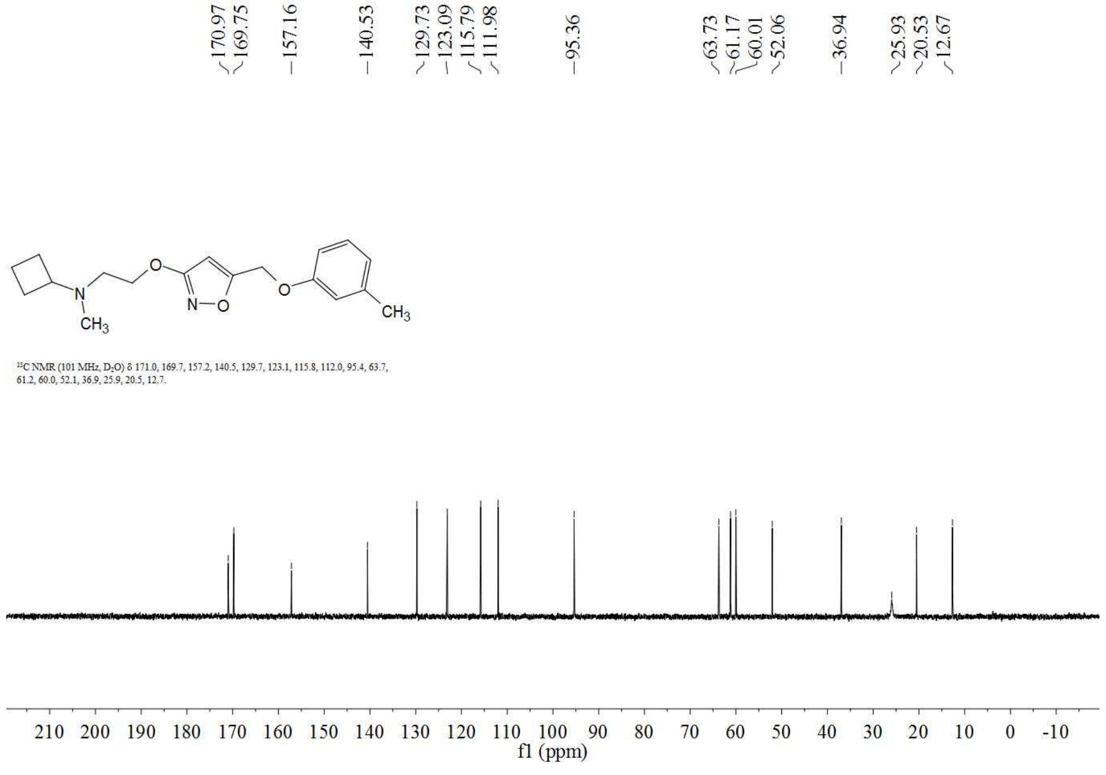

^{1}H NMR and ^{13}C NMR spectra of compound **28**

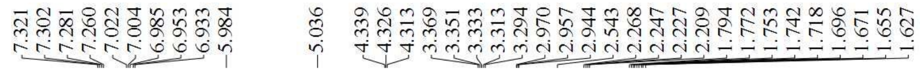

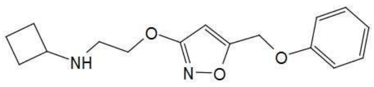

^{1}H NMR (400 MHz, $CDCl_3$) δ 7.30 (t, J = 8.0 Hz, 2H), 7.00 (t, J = 7.4 Hz, 1H), 6.94 (d, J = 8.0 Hz, 2H), 5.98 (s, 1H), 5.04 (s, 2H), 4.33 (t, J = 5.2 Hz, 2H), 3.44 – 3.26 (m, 1H), 2.96 (t, J = 5.2 Hz, 2H), 2.54 (br, 1H), 2.32 – 2.16 (m, 2H), 1.88 – 1.51 (m, 4H).

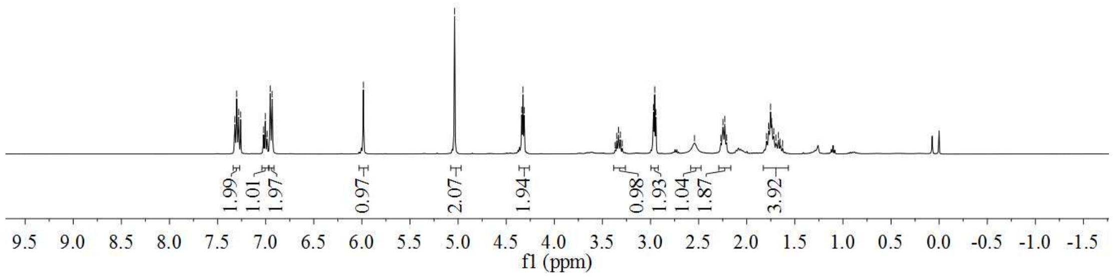

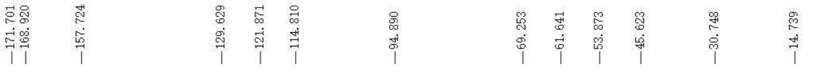

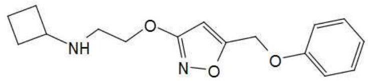

^{13}C NMR (101 MHz, Chloroform-d) δ 171.70 , 168.92 , 157.72 , 129.63 , 121.87 , 114.81 , 94.89 , 69.25 , 61.64 , 53.87 , 45.62 , 30.75 , 14.74 .

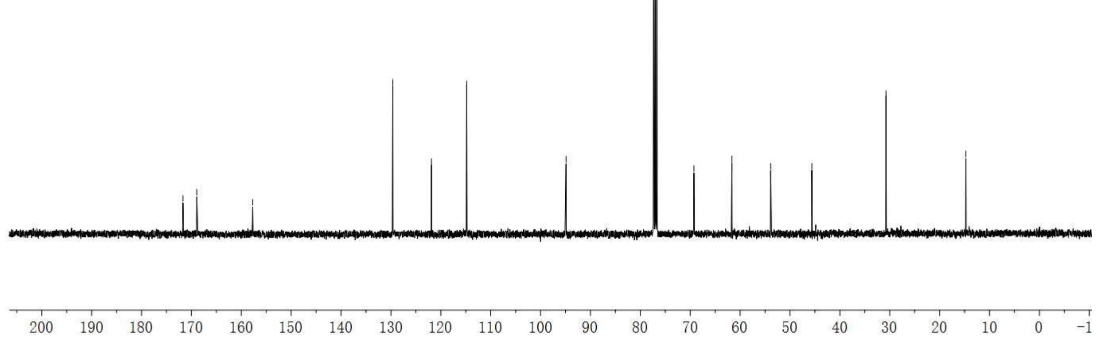

^{1}H NMR and ^{13}C NMR spectra of compound **29**

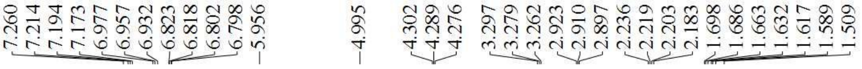

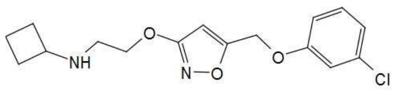

^{1}H NMR (400 MHz, $CDCl_3$) δ 7.19 (t, J = 8.2Hz, 1H), 6.97 (d, J = 8.0 Hz, 1H), 6.93 (s, 1H), 6.81 (dd, J = 8.2, 1.8 Hz, 1H), 5.96 (s, 1H), 4.99 (s, 2H), 4.29 (t, J = 4.0 Hz, 2H), 3.34 – 3.21 (m, 1H), 2.91 (t, J = 4.0 Hz, 2H), 2.26 – 2.12 (m, 2H), 1.75 – 1.57 (m, 4H), 1.51 (m, 1H).

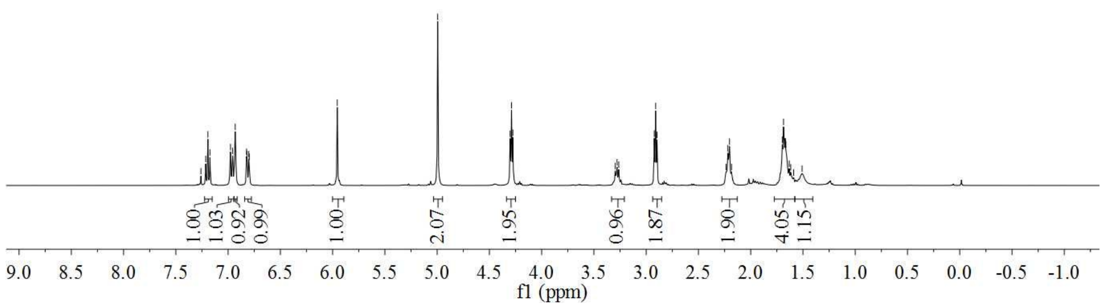

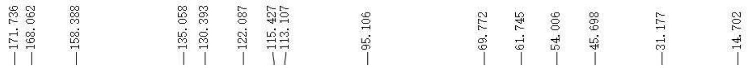

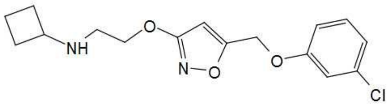

^{13}C NMR (101 MHz, Chloroform-d) δ 171.74 , 168.06 , 158.39 , 135.06 , 130.39 , 122.09 , 115.43 , 113.11 , 95.11 , 69.77 , 61.75 , 54.01 , 45.70 , 31.18 , 14.70 .

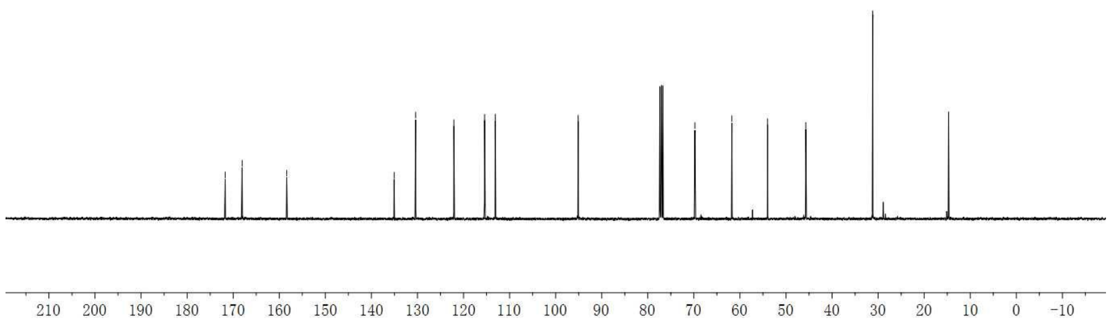

^{1}H NMR and ^{13}C NMR spectra of compound **30**

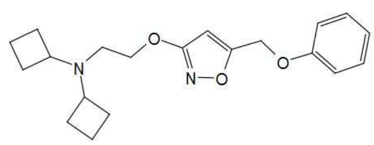

^{1}H NMR (400 MHz, $CDCl_3$) δ 7.29 (t, J = 8.0 Hz, 2H), 6.99 (t, J = 7.4 Hz, 1H), 6.94 (d, J =8.0 Hz, 2H), 5.96 (s, 1H), 5.02 (s, 2H), 4.25 (t, J = 6.2 Hz, 2H), 3.28 – 3.14 (m, 2H), 2.89 (t, J = 6.2 Hz, 2H), 2.11 – 1.85 (m, 8H), 1.72 – 1.50 (m, 4H).

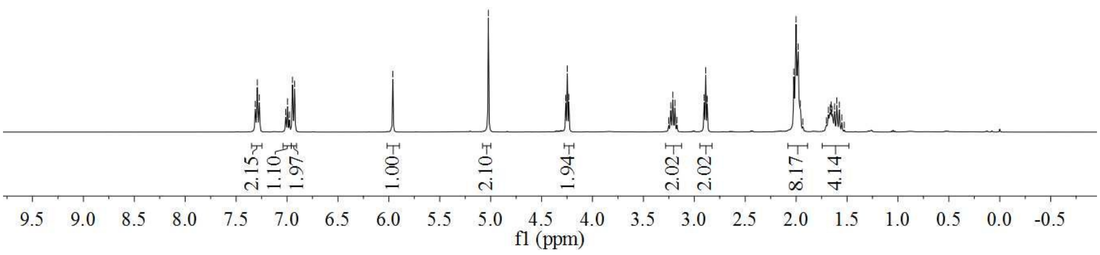

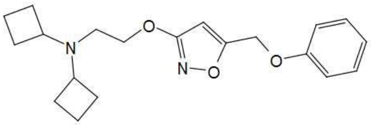

^{13}C NMR (101 MHz, Chloroform-d) δ 171.61 , 168.85 , 157.73 , 129.61 , 121.84 , 114.80 , 94.95 , 67.78 , 61.64 , 57.36 , 46.24 , 28.76 , 15.09 .

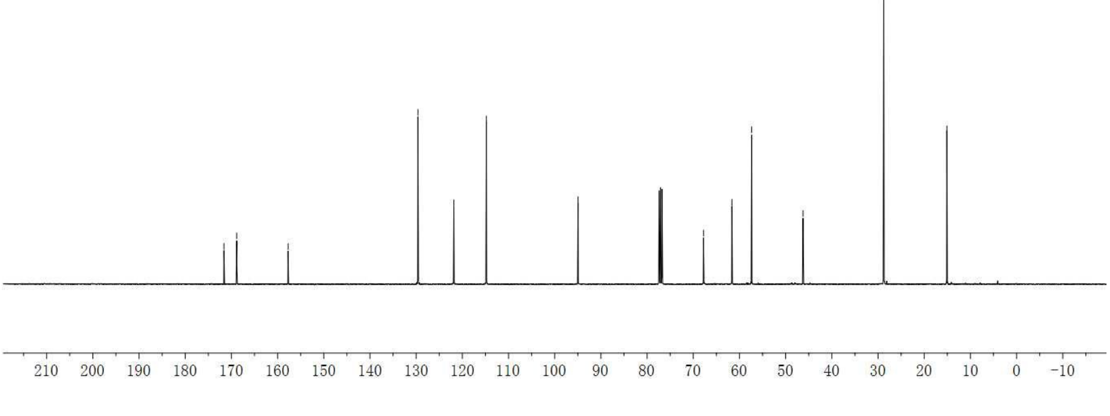

Supplement: RA-008-C8RA00072G-s001 [file RA-008-C8RA00072G-s001.pdf]
